# Precise identification of cell states altered in disease using healthy single-cell references

**DOI:** 10.1038/s41588-023-01523-7

**Published:** 2023-10-12

**Authors:** Emma Dann, Ana-Maria Cujba, Amanda J. Oliver, Kerstin B. Meyer, Sarah A. Teichmann, John C. Marioni

**Affiliations:** 1 Wellcome Sanger Institute, Wellcome Genome Campus, Cambridge, UK; 2 Theory of Condensed Matter Group, The Cavendish Laboratory, University of Cambridge, Cambridge, UK; 3 European Molecular Biology Laboratory, European Bioinformatics Institute (EMBL-EBI), Cambridge, UK; 4 Present Address: Genentech, San Francisco, CA USA

**Keywords:** Computational biology and bioinformatics, Data processing

## Abstract

Joint analysis of single-cell genomics data from diseased tissues and a healthy reference can reveal altered cell states. We investigate whether integrated collections of data from healthy individuals (cell atlases) are suitable references for disease-state identification and whether matched control samples are needed to minimize false discoveries. We demonstrate that using a reference atlas for latent space learning followed by differential analysis against matched controls leads to improved identification of disease-associated cells, especially with multiple perturbed cell types. Additionally, when an atlas is available, reducing control sample numbers does not increase false discovery rates. Jointly analyzing data from a COVID-19 cohort and a blood cell atlas, we improve detection of infection-related cell states linked to distinct clinical severities. Similarly, we studied disease states in pulmonary fibrosis using a healthy lung atlas, characterizing two distinct aberrant basal states. Our analysis provides guidelines for designing disease cohort studies and optimizing cell atlas use.

## Abstract (web summary)

In single-cell studies, combining healthy reference atlases and designed control datasets allows more precise identification of disease-associated cell states.

## Main

Precise identification of cell phenotypes altered in disease with single-cell genomics can yield insights into pathogenesis, biomarkers and potential drug targets^1–8^.

The standard approach to identify altered cell states involves joint analysis of single-cell RNA sequencing (scRNA-seq) data from diseased tissues and a healthy reference. This typically includes integrating cellular profiles from different conditions into a common phenotypic latent space to match common cell types and minimize technical differences^9,10^. Subsequently, differential analysis is performed on matched cell states between healthy and diseased cells to identify differences in gene expression patterns or cellular composition^11–15^. Regardless of the methods used for these steps, the selection of the healthy reference dataset is crucial.

**Fig. 1:**
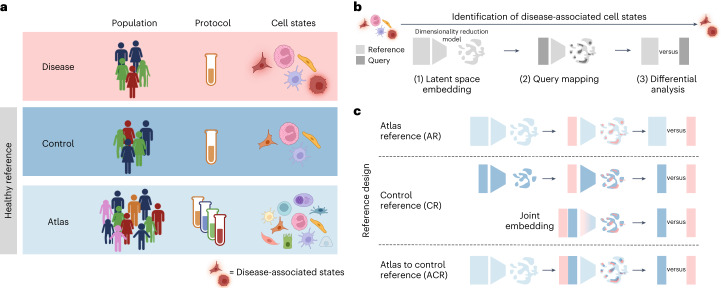
Using healthy reference datasets to discover disease-associated cell states. **a**, Schematic of attributes of disease, control and atlas datasets, with regard to population-level variation, experimental protocols and heterogeneity of cell states captured. In a disease dataset, biological samples typically originate from tens of individuals from a relatively homogeneous population (for example, recruited from the same hospital), and the same experimental protocol is used across samples for dissociation, library preparation and sequencing (or experiments are designed to minimize confounding with cohort-specific variables). We defined a healthy reference dataset as a control if it matched the disease dataset in terms of cohort characteristics and experimental protocols. We defined a reference dataset as an atlas if it aggregated data from hundreds to thousands of individuals from multiple cohorts, profiled with several experimental protocols. Typically, such integrated datasets capture a larger variety of healthy cell states compared to smaller cohorts. **b**, Schematic of the analysis workflow to detect disease-associated cell states: a dimensionality reduction model was trained on a healthy reference dataset (step 1); then, the query dataset, including the disease dataset, was mapped to the reference model with transfer learning (step 2) and finally differential analysis was performed to contrast matched cell states from healthy and disease samples. **c**, Schematic of the reference design options tested in this study, according to the workflow in **b**, using the atlas dataset as reference (light blue), the control dataset as reference (dark blue) or both. For the CR design, we compared latent embedding with query mapping (as shown in **b**) with joint embedding training a latent embedding model on the concatenated control and disease dataset. Panel **a** was created with BioRender.com.

Large-scale profiling of healthy samples by the Human Cell Atlas community has yielded large, harmonized collections of data from multiple organs, or atlas datasets (http://data.humancellatlas.org/). In tissues like lung and blood, millions of cells have been profiled from hundreds to thousands of individuals. Computational analyses allow for meaningful integration of these datasets, providing a comprehensive view of cell phenotypes in a tissue, while minimizing technical variation. Nevertheless, the characteristics of the samples included in an atlas might differ greatly from those of a disease cohort (Fig. 1a). This could introduce false discoveries if confounding factors are unknown or not appropriately handled in statistical testing. Despite this, several studies use atlas datasets as references for discovering disease states^1,16–19^, especially for tissues where obtaining matched healthy controls is challenging, such as the brain^20,21^.

In contrast, several studies collect matched control samples from healthy tissue alongside the disease samples^5,22–25^, with similar demographic and experimental protocol characteristics. This minimizes the risk of false positives driven by confounders. However, collection of a large number of healthy control samples is not always practical or possible. Moreover, using a relatively small number of samples for the integration step increases the risk of missing rare cell states and overinterpreting sample-specific noise. Understanding how features of the reference dataset impact identification of disease-associated cell states will guide effective data reuse, design of disease studies and future cell atlasing efforts.

In this study, we compare the use of atlas and control datasets as references for the identification of disease-associated cell states. We demonstrate the benefits of using an atlas dataset as reference for latent embedding and of a control dataset as reference for differential analysis, with important implications for both experimental design and use of single-cell disease cohorts.

## Results

### Reference design for disease-associated state identification

To optimize the selection of a reference dataset for the identification of disease-associated cell states, we considered the following workflow (Fig. 1b). First, a dimensionality reduction model is trained on the healthy dataset (the embedding reference dataset) to learn a latent space representative of cellular phenotypes while minimizing batch effects. Next, this model is used for transfer learning to map the query dataset, which includes the disease samples, to the same latent space^9,10^. Finally, differential analysis is performed to compare cells between disease and healthy samples (differential analysis reference) to identify disease-associated states. We defined a healthy reference dataset as a control if it matched the disease dataset in terms of cohort characteristics and experimental protocols. We defined an atlas reference (AR) dataset as one that aggregated data from hundreds to thousands of individuals from multiple cohorts, collected with several experimental protocols. With this workflow, we outlined three alternatives for selecting a reference dataset (reference design) (Fig. 1c): (1) the AR design; (2) the control reference (CR) design, where either type of healthy dataset is used as the embedding reference and as the differential analysis reference; and (3) an atlas to control reference (ACR) design, where an atlas and a control dataset are used in different steps of the workflow. In this analytical design, the atlas dataset serves as the embedding reference, while the disease and control datasets are mapped to the same latent space; finally, differential analysis is performed contrasting the disease dataset to the control dataset only. For the CR design, we compared a workflow for latent embedding where the control dataset was used as reference for query mapping, and another where the latent embedding model was trained on the concatenated control and disease datasets (Supplementary Note 2.4).

In the following sections, we quantify the ability of these three designs to identify disease-specific cell states in simulations and real data.

### Detection of out-of-reference cell states in simulations

**Fig. 2:**
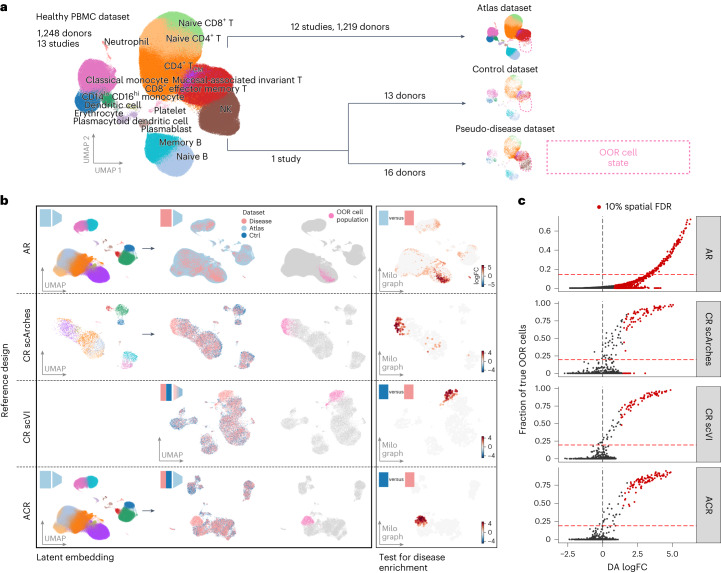
Benchmarking setup for comparison of reference designs on detection of OOR cell states. **a**, Schematic of the strategy used to simulate ground truth OOR cell states in real data from healthy human PBMCs, split into atlas (513,565 cells), control (5,671 cells) and pseudo-disease (7,505 cells) datasets. We tested simulations alternatively using 15 annotated cell states as out-of-reference (OOR) cell states. **b**, Example outcome of latent embedding and differential analysis with different reference designs. Left, uniform manifold approximation and projection (UMAP) embedding of scVI latent space learned on the embedding reference dataset. Points are colored according to cell type clusters (as in **a**); the icons in the top left corner indicate the type of embedding reference dataset used. Center, UMAP embedding of cells from the differential analysis reference and disease datasets on scVI latent space learned from the embedding reference dataset, colored according to type of dataset and to highlight (in pink) the OOR cell state. For the CR design, we differentiated between latent embedding with query mapping (CR scArches) and embedding in one step, training an scVI model on the concatenated control and disease dataset (CR scVI). Right, Milo neighborhood graph visualization of DA testing results: each point represents a neighborhood, and points are colored according to the log fold change (logFC) in cell abundance between disease and reference cells. Only neighborhoods where significant enrichment in disease cells (10% spatial FDR and log fold change > 0) was detected are colored. Points are positioned based on the coordinates in the UMAP embedding of the neighborhood index cell; the size of points is proportional to the number of cells in the neighborhood. The horizontal dashed lines are used to separate the phases of the workflow. **c**, Scatterplot of DA log fold change against the fraction of disease-specific cells for each neighborhood for the simulation shown in **c**. Each plot represents a different reference design. Colored points indicate neighborhoods where significant enrichment in disease cells (10% spatial FDR and log fold change > 0) was detected. The vertical line is 0; the horizontal line is the threshold to consider the neighbourhood as a true positive.

To test a scenario with ground truth, we simulated the attributes of atlas, control and disease datasets by splitting scRNA-seq data from 13 studies that profiled healthy peripheral blood mononuclear cells (PBMCs) from 1,248 donors (Supplementary Table 1 and Methods). We selected one study and randomly split the donors to simulate a pseudo-disease and a control dataset (Fig. 2a). This ensured that cohort demographics and experimental protocols were matched, preserving donor and library effects present in real data. The remaining cells (1,219 donors) form the atlas dataset. To simulate a cell population specific to the pseudo-disease dataset, hereafter an out-of-reference (OOR) state, we selected one or more annotated cell types and removed cells with those labels from the control and atlas datasets.

To identify the OOR state, we first learned a latent space embedding on the chosen reference (atlas or control) using single-cell variational inference (scVI)^26^ (Fig. 2b, left). Then, we used transfer learning with scArches^9^ to map the query dataset(s) to the trained scVI model. For the CR design with joint embedding (CR scVI), we trained the scVI model on the concatenated pseudo-disease and control datasets (Fig. 2b, center). In the ACR design, the atlas dataset was used to train the latent embedding model; however, after mapping with scArches, only the disease and control datasets are considered. Finally, we used neighborhood-level differential abundance (DA) testing with Milo^11^ to identify cell states enriched in the disease dataset (Fig. 2b, right).

**Fig. 3:**
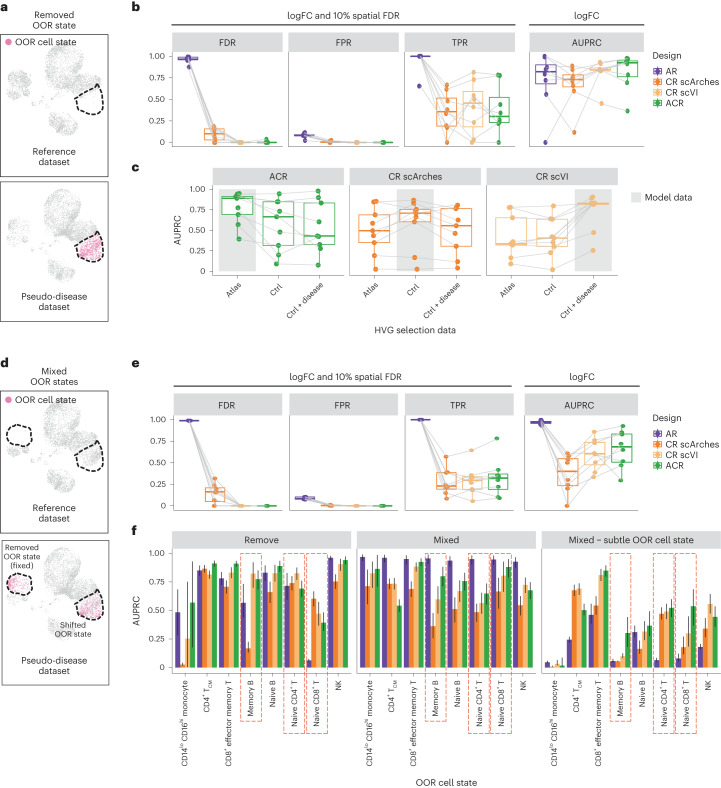
Detection of simulated OOR cell states. **a**, Illustration of removed OOR state perturbation. The dashed outlines denote the position of the OOR cell state. **b**, Performance comparison of reference designs in the detection of OOR cell states. To compare performance considering the log fold change and confidence (10% spatial FDR), we measured the FDR, FPR and true positive rate (TPR). To compare performance using the log fold change only as a metric for prioritization, we measured the AUPRC. The points represent simulations with different OOR states (eight states, excluding simulations where fewer than 250 OOR cells were present after splitting the pseudo-disease and control dataset). Tests on the same simulated data are connected. **c**, Box plots of AUPRC to detect OOR cell states with embedding models trained on different sets of 5,000 HVGs, selecting HVGs in the atlas dataset, in the control dataset or in the concatenated control and pseudo-disease datasets (control + disease). The color represents different reference designs. Tests on the same simulated data are connected. The gray box denotes the type of data used to train the model for each design. **d**, Illustration of mixed OOR state perturbation: all simulations have a fixed cell state removed from the control and atlas datasets (classical monocytes) and a varying shifted OOR cell state, where cells of the OOR cell state are split in two groups based on principal component analysis (PCA), and only one group is removed from the atlas and control datasets (shifted OOR state). **e**, As in **b**, but with mixed OOR states. **f**, Bar plots of the AUPRC for OOR state detection with different types of perturbation on the same OOR cell state, colored according to reference design. The rightmost plot shows the AUPRC for the detection of the shifted OOR cell state, excluding the fixed removed state. The height of the bar denotes the AUPRC computed on real data. The error bars indicate the 95% confidence interval (CI) calculated from bootstrapping with 1,000 resampling iterations. Cases where the CR design outperformed the ACR design when only the OOR state is removed are highlighted by the red dashed rectangles. In all box plots, the center line denotes the median; the box limits denote the first and third quartiles; and the whiskers denote 1.5× the interquartile range (IQR).

**Extended Data Fig. 1:**
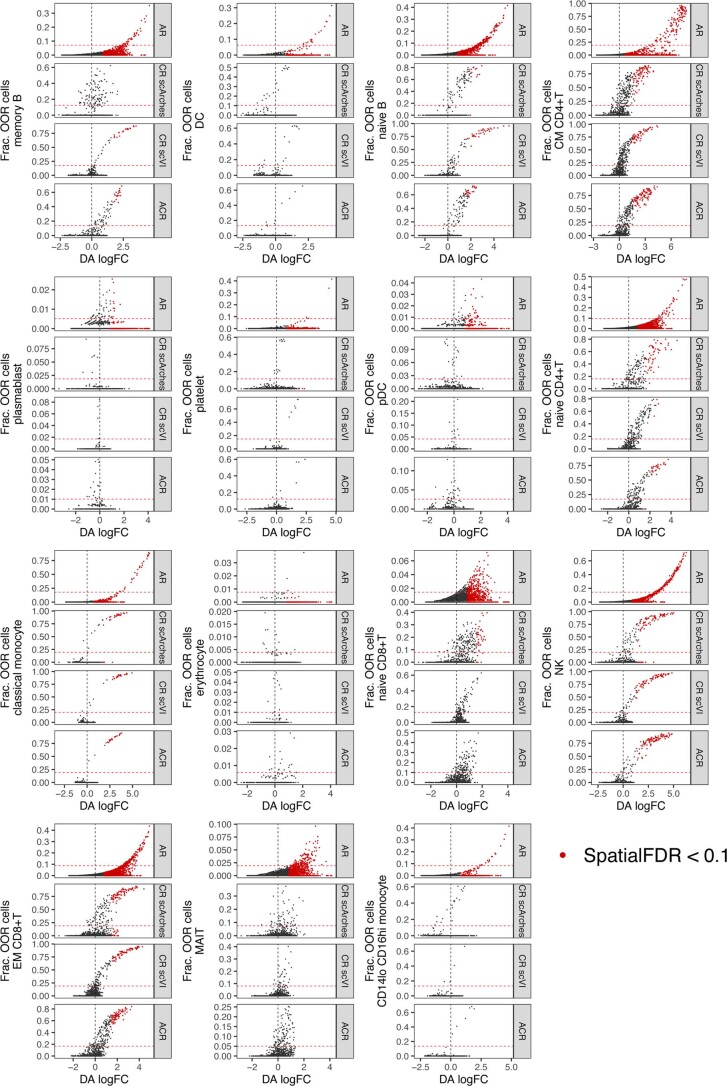
Out-of-reference recovery across simulations. Scatterplot of differential abundance log-Fold Change (DA logFC) against fraction of out-of-reference (OOR) cells for each neighbourhood, in simulations with different removed OOR cell populations (indicated in y-axis). Colored points indicate neighbourhoods where the enrichment was significant (10% SpatialFDR, logFC > 0). The dotted red line indicates the threshold used to define true positives for precision-recall analysis (20% of the higher fraction in the simulation).

**Extended Data Fig. 2:**
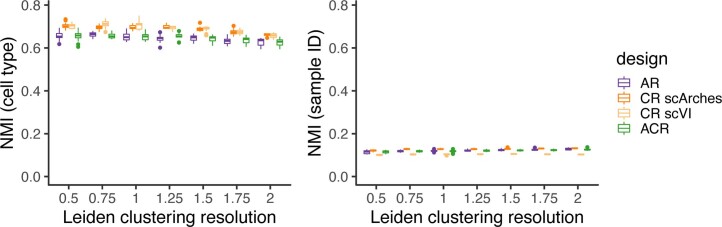
Batch correction and biological conservation with latent dimensions learnt with different reference design. Quantification of overlap between cell type labels (as a measure of biological conservation, left) and sample IDs (as a measure of batch effect, right) and clusters of disease cells on latent dimensions after scArches mapping with different designs (color). The overlap between clusters and covariates is measured by the Normalised Mutual Information (NMI), using the implementation in scikit-learn v1.1.2 (ref. ^74^). Each box plot shows the median and interquartile range for simulations with different OOR cell populations (n = 15 simulations). NMI values for leiden clustering with increasing resolution (x-axis) are shown. In boxplots the center line denotes the median; box limits, first and third quartiles; whiskers, 1.5X interquartile range.

**Extended Data Fig. 3:**
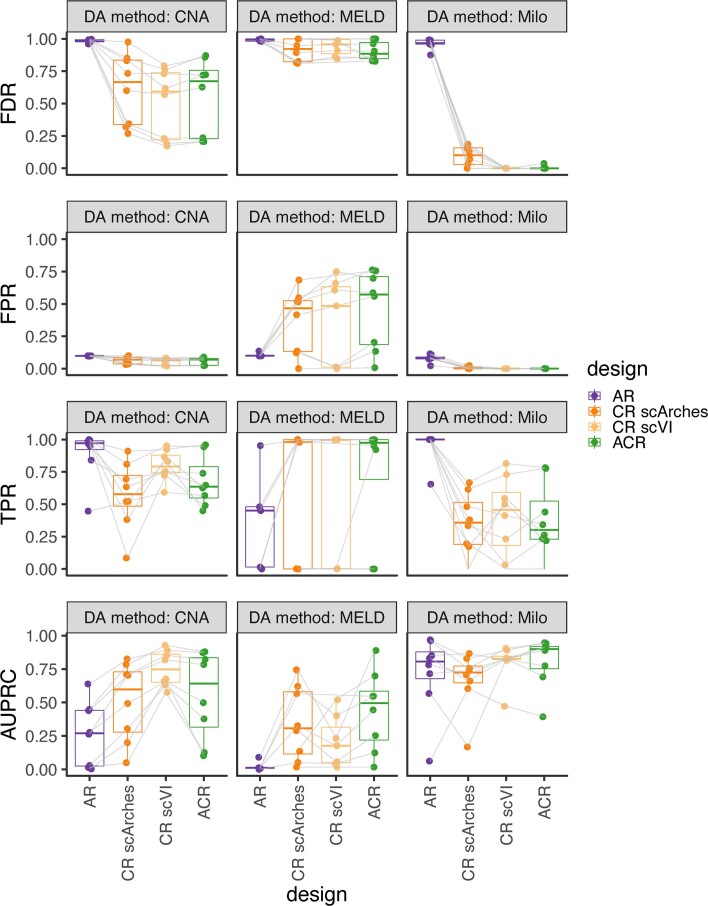
Reference design comparison with alternative differential analysis methods for OOR detection. Boxplots of false discovery rate (FDR), false positive rate (FPR), true positive rate (TPR) and Area Under the Precision-Recall Curve (AUPRC) for detection of OOR cell states with different reference designs (boxplot colour) using 3 different methods for differential cell abundance analysis: co-varying neighbourhood analysis (CNA), MELD and Milo. Points represent simulations with different OOR populations (n = 8, selecting OOR states with at least 250 cells). Tests on the same simulated data are connected. In boxplots the center line denotes the median; box limits, first and third quartiles; whiskers, 1.5X interquartile range.

**Extended Data Fig. 4:**
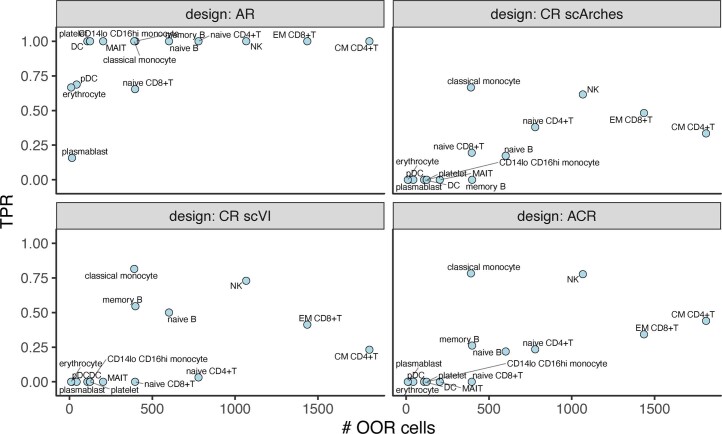
Statistical power is dependent on the size of the OOR cell state across reference designs. Scatterplot of number of cells in the simulated OOR state (x-axis) against the true positive rate (TPR, y-axis) of OOR state detection with alternative reference designs.

We first considered a scenario where a single-cell-type cluster is selected as the OOR state and removed from the healthy references (Fig. 3a). Across simulations with different OOR states, we observed that using the combination of the atlas and control datasets (ACR design) led to sensitive detection of neighborhoods with a high fraction of OOR cells (Figs. 2c and 3b, and Extended Data Fig. 1). Conversely, the AR design led to an inflated number of false positives, where significant enrichment was also detected when the fraction of unseen cells was low or 0. Using only the control dataset, latent embedding with query mapping led to more balanced log fold changes, but still a higher false discovery rate (FDR) than the ACR design, while performance with a joint embedding was comparable to the ACR design. Notably, we found minimal difference in the quality of integration with different designs (Extended Data Fig. 2). The difference between reference design results was also consistent when applying alternative methods for DA analysis^13,15^ (Extended Data Fig. 3). Finally, as expected, the power to detect OOR cell states depended, for all methods, on the number of cells present, with a minimum of 250 cells per cell type needed to identify the OOR population (Extended Data Fig. 4).

We hypothesized that the good performance in OOR state detection with the CR scVI design could be explained by feature selection. Latent embedding models are trained on the top 5,000 highly variable genes (HVGs) in the input dataset (Methods). When training on concatenated disease and control datasets, marker genes for the OOR population are more likely to be among the HVGs. We compared the performance of different reference designs trained using HVGs from the atlas dataset, from the control dataset or from the concatenated control and disease dataset. For all designs, the area under the precision-recall curve (AUPRC) for OOR state detection was highest when using HVGs selected on the same data used to train the model. However, only the CR design with joint embedding showed a substantial decrease in performance when selecting HVGs without using the disease dataset (Fig. 3c). On average, 81% of the HVGs selected from the control and disease data were shared with the set selected from control only and 68% were shared with the set selected from the atlas only. These results indicate that the performance of joint embedding with CR design is sensitive to the feature selection strategy used to train the latent embedding model.

We reasoned that this might impact performance when multiple transcriptionally distinct OOR states are present in the disease population. To test this, we conducted simulations where we removed a fixed cell population (corresponding to classical monocytes) from the reference datasets and then defined a second variable OOR cell state (shifted OOR state) by splitting a cell type population into two distinct groups (Methods and Fig. 3d). The ACR design performed best in OOR state identification (Fig. 3e). In particular, in all simulations where the CR scVI design outperformed the ACR design when the OOR state was removed, the ACR design could distinguish better OOR states in the mixed case, even when considering only recovery of the shifted OOR state (Fig. 3f). In one case (simulation with CD4^+^ central memory T (T_CM_) cells as shifted OOR state), we observed a significant drop in performance with the ACR design if the OOR state was shifted instead of removed.

In summary, differential analysis using control datasets drastically reduced the rate of false discoveries in the detection of disease-associated cell states. Of note, studies using atlas datasets to identify disease-associated states^9,10,16^ might use criteria different from DA to detect OOR cells, such as distance in the latent space, label transfer uncertainty or differential expression analysis. We compared these alternatives to our workflow in Supplementary Note 2.1.

### Robustness of OOR detection with the ACR design

**Fig. 4:**
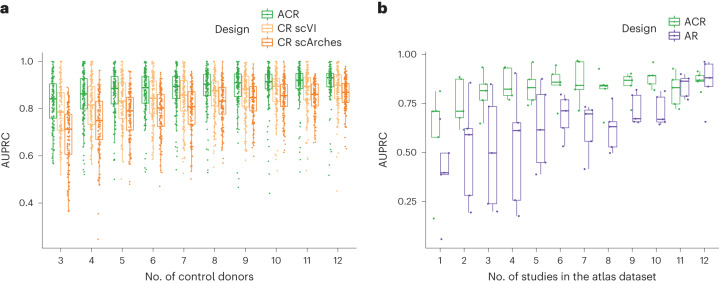
Robustness of detection of OOR state with the ACR design. **a**, Robustness to the size of the control cohort with the ACR and CR designs. Box plots of the AUPRC for simulations with an increasing number of donors in the control dataset (*x* axis), using the ACR (green) or CR design with query mapping (CR scArches, orange) or joint embedding (CR scVI, yellow). The results from simulations with five different OOR cell states, selected according to top mean TPR across designs in Fig. 3b and using five different samples of donors for each number of control donors and OOR state, are shown. In these simulations, five, seven or nine donors were used in the disease dataset (see Supplementary Fig. 1 for the full breakdown). **b**, Robustness to the size of the atlas dataset with the ACR and AR designs. Box plots of the AUPRC for simulations with an increasing number of studies in the atlas dataset, using the ACR (green) or AR (purple) design. The results from simulations with five different OOR cell states, selected according to top mean TPR across designs in Fig. 3b, are shown (see Supplementary Fig. 2 for a full breakdown).

We next assessed the robustness of different reference designs to heterogeneity in the control and atlas datasets. We first tested whether using the atlas reduces the number of control donors needed to detect disease-specific states by simulating control datasets of increasing size (Methods). While sensitivity declined for all designs when using a very small control cohort, the ACR design maintained the highest performance in OOR state detection compared to the CR design, regardless of the latent embedding strategy (*t*-test *P* < 0.01 for AUPRC distributions across control cohort sizes for both the CR scVI and CR scArches designs) (Fig. 4a and Supplementary Fig. 1). The difference in performance was especially marked when simulating a smaller disease cohort (Supplementary Fig. 1). These results suggest that using the ACR design can minimize the number of control samples required. In Supplementary Note 2.3, we tested options for cases where collecting matched control samples is not feasible.

We also tested how OOR state detection was affected by variation in the atlas dataset. We first confirmed robustness to removal of any given study from the atlas dataset (Supplementary Note 2.2.1). Then, we measured performance with the AR and ACR designs when including an increasing number of PBMC studies in the atlas dataset (Methods). While the results were always significantly affected when using just one or two studies as the atlas dataset, sensitivity with the ACR design was stable when the atlas included at least 10,000 cells (Fig. 4b and Supplementary Fig. 2). Without controls, we observed a stronger dependency of performance with atlas size (Pearson correlation of AUPRC and size: *R*^2^ = 0.69, *P* = 7.2 × 10^−7^ for the AR design; *R*^2^ = 0.4, *P* = 0.0017 for the ACR design). Notably, the false positive rate (FPR) increased with smaller atlas datasets with an AR design (Supplementary Fig. 2). We compared the use of a cross-tissue or tissue-specific atlas for the ACR design (Supplementary Note 2.2.2), as a practical alternative where the availability of tissue-specific data might be scarce.

In summary, combining the use of an atlas and control dataset led to robust detection of putative disease states, even with a varying quality of the control or atlas dataset.

### Detection of interferon-stimulated states in patients with coronavirus disease 2019

**Fig. 5:**
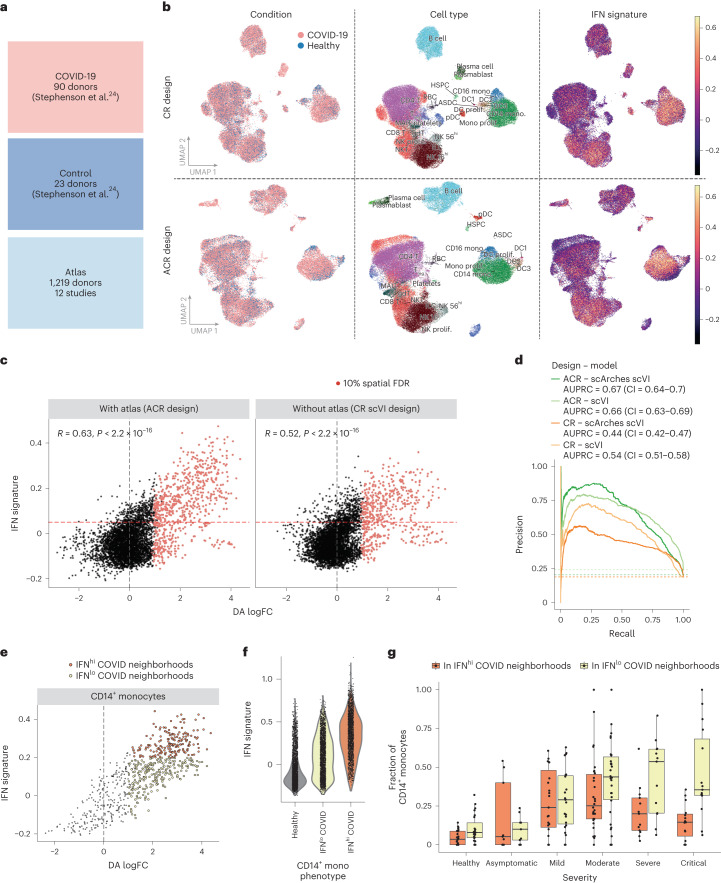
Detection of cell states associated with COVID-19 in a case-control cohort with a healthy atlas. **a**, Overview of composition of disease (48,083 cells), control (14,426 cells) and atlas dataset (513,565 cells). **b**, UMAP embedding of cells from the COVID-19 and healthy datasets integrated with a CR (joint embedding, top) or ACR (bottom) design. Cells are colored according to disease condition (left), broad annotated cell type (middle) and expression of IFN signature (right). Mono, monocyte; prolif., proliferative; RBC, red blood cell; T_reg_, regulatory T. **c**, Scatterplot of neighborhood DA log fold change against the mean expression of IFN signature with the ACR (left) and CR (right) designs. Neighborhoods where enrichment in COVID-19 cells was significant (log fold change > 0 and 10% spatial FDR) are colored. Pearson correlation coefficients and *P* values for the significance of the correlation are reported (two-sided test). **d**, Precision–recall curves for the detection of IFN-activated neighborhoods with DA log fold change for alternative designs (ACR or CR) and using joint embedding of reference and disease datasets (scVI) or transfer learning (scArches scVI). The AUPRC is reported in the legend, with the 95% CI calculated from bootstrapping with 1,000 resamplings shown in brackets. The dashed lines denote the baseline value for the AUPRC, indicating the case of a random classifier. **e**, Scatterplot of neighborhood DA log fold change against the mean expression of IFN signature with the ACR design for neighborhoods of CD14^+^ monocytes. The colored points indicate neighborhoods where the enrichment in COVID-19 cells was significant (10% spatial FDR). Neighborhoods are colored according to IFN phenotype. **f**, Distribution of IFN signature score for cells belonging to neighborhoods assigned to three alternative CD14^+^ phenotypes. **g**, Distribution of COVID-19-enriched CD14^+^ phenotypes across patients with varying disease severity (healthy: *n* = 23 patients; asymptomatic: *n* = 9 patients; mild: *n* = 23 patients; moderate: *n* = 30 patients; critical: *n* = 15 patients; severe: *n* = 13 patients). Each point represents a donor; the *y* axis shows the fraction of all CD14^+^ monocytes in that donor showing an IFN^hi^ COVID-19-enriched phenotype (orange) and an IFN^lo^ COVID-19-enriched phenotype (yellow). The remaining fraction represents monocytes with a healthy phenotype (not shown). In the box plots, the center line denotes the median; the box limits denote the first and third quartiles; and the whiskers denote 1.5× the IQR.

We next assessed the benefits of using a healthy atlas to identify altered states in a real patient cohort. We used a published scRNA-seq dataset of PBMCs from 90 patients with varying severities of coronavirus disease 2019 (COVID-19) and 23 healthy volunteers^24^. As an atlas dataset, we used harmonized scRNA-seq profiles from 12 studies involving 1,219 healthy individuals (Fig. 5a). We compared the use of the healthy PBMC atlas for latent embedding (ACR design) against using only the COVID-19 and control datasets with joint embedding (CR design). To quantify the ability of different designs to identify disease-associated states, we tested whether cells expressing genes involved in interferon (IFN) signaling, a key antiviral response pathway and a recognized hallmark of COVID-19, could be detected among the COVID-19-enriched neighborhoods (Fig. 5b and Methods).

**Extended Data Fig. 5:**
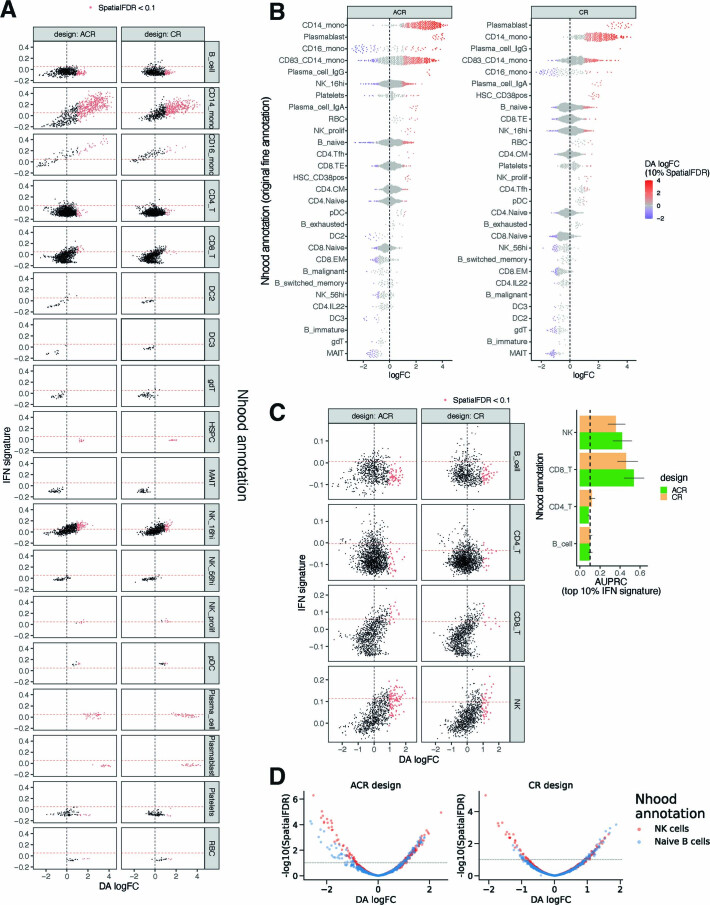
Reference design comparison on COVID-19 cohort. (**a**) Scatterplot of neighbourhood differential abundance log-Fold Change (DA logFC) against the mean expression of IFN signature with ACR design (left) and CR design with joint embedding (right), stratified by cell type annotation. Colored points indicate neighbourhoods where the enrichment was significant (10% SpatialFDR and logFC > 0). The dotted line denotes the threshold for high-IFN used for precision-recall analysis. **(b)** Beeswarm plot of DA logFC annotating neighbourhoods by fine annotation by Stephenson et al. Neighbourhoods where the differential abundance was significant (10% SpatialFDR) are colored. Annotations are ordered by the value of the maximum logFC for the annotation, to visualize which cell types are prioritized for each design. **(c)** (left) As in (A) but close-up on lymphoid cell types. The red dotted line denotes the 90% quantile of mean IFN signature, used to identify the top 10% IFN-high states for each lymphoid cell type for precision-recall analysis. (right) Area under the precision-recall curve for identification of top 10% IFN-high neighbourhoods in lymphoid cell types. The dotted line denotes the baseline value for the AUPRC, indicating the case of a random classifier. Error bars denote the 95% confidence interval of AUPRC calculated from bootstrapping with 1000 resamplings. The height of the bar denotes the AUPRC computed on the real data. (**d**) Volcano plot for differential abundance analysis on neighbourhoods of NK cell neighbourhoods (CD16hi NK cells and proliferating NK cells) and naive B cell neighbourhoods. The dotted line denotes the significance threshold of 10% SpatialFDR.

The ACR design showed a stronger correlation between DA log fold change and the mean IFN signature (ACR Pearson *R* = 0.63, CR Pearson *R* = 0.52, Fisher’s *z*-transformation *P* < 2.2 × 10^−16^), indicating better prioritization of IFN^hi^ cell states (Fig. 5c), regardless of the latent embedding strategy used (Fig. 5d). Stratifying according to cell type, the correlation was especially strong in myeloid cells, where the strongest IFN stimulation was observed (Extended Data Fig. 5a). Among the IFN^lo^ states prioritized with the ACR design, we found primarily plasmablasts and plasma cells (Extended Data Fig. 5b), followed by platelets, all expected to expand in COVID-19 (refs. ^27,28^). For lymphocytes, where the average expression of IFN genes was lower than in myeloid cells, the ACR design outperformed the CR design in prioritizing the top 10% IFN^hi^ neighborhoods in natural killer (NK) and CD8^+^ T cells, while neither design distinguished IFN^hi^ CD4^+^ T cells or B cells (Extended Data Fig. 5c). The CR design prioritized IFN^lo^ naive B cells over other IFN^hi^ subsets, such as CD16^hi^ and proliferating NK cells (Extended Data Fig. 5b–d), contradicting the widely reported lymphopenia in patients with COVID-19 (ref. ^29^).

**Extended Data Fig. 6:**
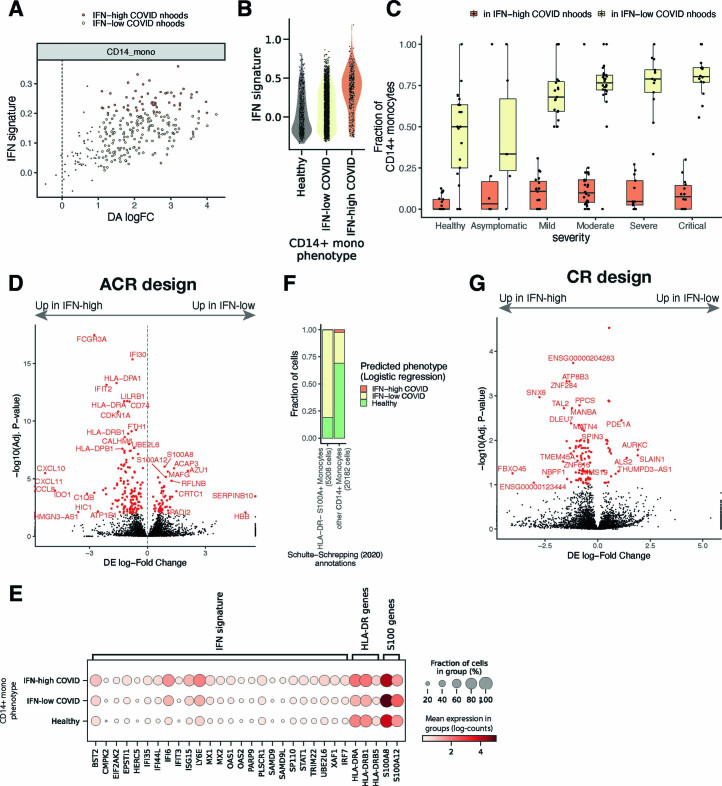
Heterogeneity in COVID-19 associated CD14+ monocyte states. (**a**) Scatterplot of neighbourhood differential abundance log-Fold Change (DA logFC) against the mean expression of IFN signature with CR design for neighbourhoods of CD14+ monocyte cells (as in Fig. 5e). (**b**) Distribution of IFN signature score for cells belonging to neighbourhoods in CR design assigned to 3 alternative CD14+ phenotypes. (**c**) COVID-19 enriched CD14+ phenotypes (from CR design) across patients with varying disease severity (Healthy: n = 23 patients, Asymptomatic: n = 9 patients, Mild: n = 23 patients, Moderate: n = 30 patients, Critical: n = 15 patients, Severe: n = 13 patients): each point represents a donor, the y-axis shows the fraction of all CD14+ monocytes in that donor showing IFN-high COVID-19 enriched phenotype (orange), and IFN-low COVID-19 enriched phenotype (yellow). The remaining fraction are monocytes with healthy phenotype (not shown). In boxplots the center line denotes the median; box limits, first and third quartiles; whiskers, 1.5X interquartile range. (**d**) Volcano plot of differential expression analysis results from comparison between IFN-high and IFN-low COVID-19 specific CD14+ phenotypes identified with ACR design. For each tested gene, the x-axis shows the logFC of the edgeR quasi-likelihood differential expression test^64^ and the y-axis shows the Benjamini-Hochberg adjusted p-value. Genes with significant DE at FDR < 1% are colored in red. A subset of significant genes with absolute logFC > 0.75 are labelled. (**e**) Dotplot of mean expression of IFN signature genes, HLA-DR genes and S100 genes for different CD14+ monocyte states identified with ACR design. Dot size is proportional to the fraction of cells expressing the gene in a group. (**f**) Predicted CD14+ monocyte phenotype for monocytes of COVID-19 patients from the Schulte-Schrepping^23^ dataset. A logistic regression model was trained on the monocytes from the Stephenson dataset^24^, and predicted phenotypes for all CD14+ monocytes in the Schulte-Schrepping^23^ dataset. The barplot shows the proportion of cells with a predicted phenotype for HLA-DRlo S100hi monocytes and for all other monocytes. (**g**) Volcano plot of differential expression analysis results from comparison between IFN-high and IFN-low COVID-19 specific CD14+ phenotypes identified with CR design (as in (D)).

Through iterative dataset subsetting, subclustering and differential analysis, several COVID-19 scRNA-seq studies distinguished IFN-stimulated COVID-19-associated subclusters and normal IFN^lo^ subtypes across immune cell types^22,30^. Yet, IFN activation is not global, and transitional or alternative pathological phenotypes might be present in COVID-19 PBMCs. In our neighborhood-level analysis with the ACR design, we observed neighborhoods with a relatively low IFN signature that were significantly associated with the disease, notably among classical (CD14^+^) monocytes (Fig. 5e). We categorized CD14^+^ monocytes into three phenotypes: normal classical monocytes; COVID-associated IFN^lo^ monocytes; and COVID-associated IFN^hi^ monocytes (Fig. 5f). The proportion of CD14^+^ monocyte phenotypes changed significantly with different disease severity: the IFN^hi^ state was most prominent in mild and asymptomatic cases compared to healthy cases (Wilcoxon test *P* = 1.19 × 10^−7^), while the IFN^lo^ state was predominant in patients with moderate-to-critical disease (Fig. 5g). This supports the notion that IFN stimulation acts as a protective pathway in the acute phase of infection^31^. Conversely, when using the CR design to define IFN^hi^ and IFN^lo^ states after differential analysis, we found a high fraction of IFN^lo^ COVID-enriched monocytes in healthy and asymptomatic individuals, indicating that this design failed to distinguish IFN^lo^ normal monocytes from the IFN^lo^ phenotype in severe COVID-19 (Extended Data Fig. 6a–c). Additionally, the fraction of IFN^hi^ cells in mild and moderate cases was not significantly higher than in severe cases (Wilcoxon test *P* = 0.325743). Differential expression analysis between IFN^hi^ and IFN^lo^ COVID-associated monocytes showed that IFN^hi^ monocytes showed higher expression of *HLA* genes, leukocyte-recruiting chemokines (*CCL8*, *CXCL10*, *CXCL11*) and markers of activation (*FCGR3A*) (Extended Data Fig. 6d,e and Supplementary Table 2). Conversely, the IFN^lo^ monocytes enriched in severe disease overexpressed *S100A* genes, previously identified as key markers of COVID-19 severity^30,32^. This *HLA-DR*^lo^ *S100A*^hi^ phenotype corresponds to a subset of dysfunctional monocytes associated with severe COVID-19, previously described in an independent cohort through direct comparison of mild and severe cases^23^ (Extended Data Fig. 6f). These markers were not recovered when comparing IFN^lo^ and IFN^hi^ COVID-19 monocytes defined by the CR design (Extended Data Fig. 6e and Supplementary Table 3).

### Detection of aberrant cell states in pulmonary fibrosis

**Fig. 6:**
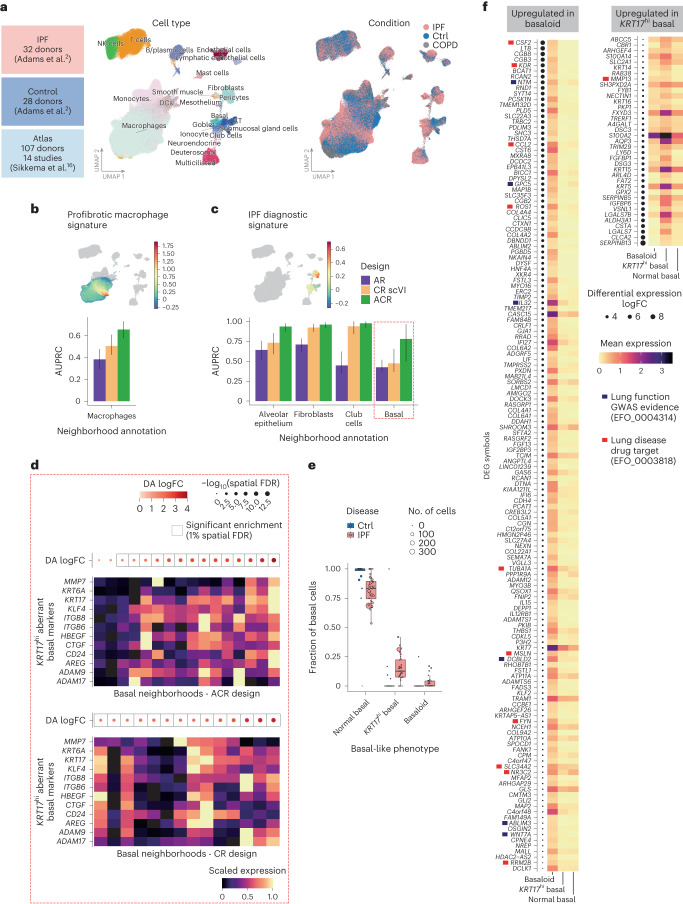
Detection of cell states associated with IPF. **a**, Overview of the composition of IPF (144,404 cells), control (95,303 cells) and atlas dataset (584,844 cells) (top) and UMAP embedding of cells from the IPF and control datasets integrated with an ACR design (bottom). Cells are colored by broad cell type annotation (left) and disease condition (IPF and COPD) (right). **b**, Detection of profibrotic macrophages with alternative reference designs. Top, UMAP embedding colored according to the scaled expression of profibrotic macrophage signature in macrophage cell compartment. Bottom, bar plot of the AUPRC for the detection of macrophage neighborhoods with high mean profibrotic signature, colored according to reference design. Bar height indicates the AUPRC computed on real data; the error bars indicate the 95% CI from bootstrapping with 1,000 resamplings. **c**, As in **b**, but for the detection of the IPF diagnostic gene signature in stromal and epithelial cells. The red dashed rectangle highlights basal cells used for follow-up analysis in **d**. **d**, Min–max scaled mean expression of marker genes for *KRT17*^hi^ aberrant basal cells (defined by Jaeger et al.^40^) in basal neighborhoods identified with the ACR design (left) and the CR design (right). Neighborhoods are ordered by increasing DA log fold change between IPF and control cells. The dots at the top indicate the log fold change (color) and spatial FDR (size) for the DA test. The boxes denote neighborhoods where the enrichment in IPF cells was significant (spatial FDR 1%). **e**, Fraction of basal-like cells of different phenotypes in samples from patients with IPF and controls (control: *n* = 28 patients; IPF: *n* = 32 patients). Each point represents a patient. Point size is proportional to the number of cells of a given phenotype. In the box plots, the center line denotes the median; the box limits denote the first and third quartiles; and the whiskers denote 1.5× the IQR. **f**, Mean expression of newly identified marker genes for aberrant basal-like phenotypes, identified by differential expression analysis between basaloid cells and *KRT17*^hi^ aberrant basal cells (Methods; see Supplementary Fig. 4 for a heatmap, including known marker genes). Genes are ordered by log fold change. The blue boxes mark genes significantly associated with genome-wide association study (GWAS) variants for lung function. The red boxes mark validated target genes of drugs in trials for lung disease.

To assess the benefit of using atlas and control datasets in other biological contexts, we analyzed a published scRNA-seq dataset of lung parenchyma samples from 32 patients with idiopathic pulmonary fibrosis (IPF), a progressive lung disease with limited treatment options, which is characterized by extracellular matrix (ECM) deposition, inflammation and scarring^33,34^. This study included data from 28 control donors and 18 patients with chronic obstructive pulmonary disease (COPD)^2^. As an atlas dataset, we used the core Human Lung Cell Atlas (HLCA) dataset^16^ (Fig. 6a).

**Extended Data Fig. 7:**
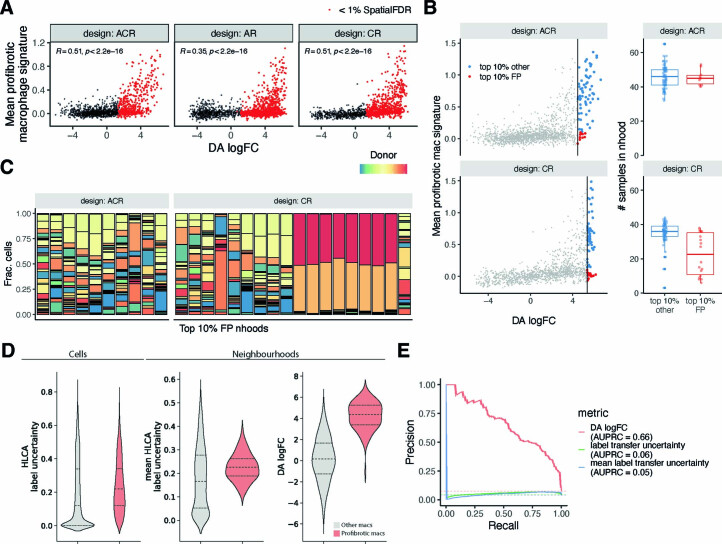
Detection of profibrotic (SPP1hi) macrophages with alternative reference designs. (**a**) Scatterplots of differential abundance log-Fold Change (DA logFC) against the mean expression of profibrotic macrophage signature in macrophage cell neighbourhoods with ACR design (left), AR design (middle) and CR design (right). Coloured points indicate neighbourhoods where the enrichment was significant (1% SpatialFDR and logFC > 0). Pearson’s correlation coefficients and p-values for significance of the correlation are reported (two-sided test). (**b**) Analysis of top 10% macrophage neighbourhoods prioritized by DA logFC using ACR and CR designs. When examining prioritized neighbourhoods with low expression of profibrotic signature (top 10% false positives), we found that with the CR design these neighbourhoods include cells from significantly less samples compared to the true positives. On the left, we mark neighbourhoods that are considered top 10% (colored), separating out False Positive (FP) neighbourhoods, where the mean profibrotic macrophage signature was below the threshold of the 90% quantile used for precision-recall analysis. The boxplots on the right show the number of samples represented in each top 10% neighbourhood (ACR other: n = 10 neighbourhoods; ACR FP: n = 65 neighbourhoods; CR other: n = 66 neighbourhoods; CR FP: n = 18 neighbourhoods). In boxplots the center line denotes the median; box limits, first and third quartiles; whiskers, 1.5X interquartile range. (**c**) Barplots of fraction of cells from each donor in top 10% false positive neighbourhoods with ACR (left) and CR design (right). (**d**) Detection of profibrotic macrophages with label transfer uncertainty score from Sikkema et al. 2022. Violin plots show the distribution of label uncertainty on cells (left), mean label uncertainty on neighbourhoods (centre) and DA logFC with ACR design for profibrotic macrophages (profibrotic macrophage signature > 90% quantile, in pink) and other macrophages (in grey). The dotted lines denote the median value and inter-quartile range. (**e**) Precision-recall curve for detection of profibrotic macrophages with metrics shown in D. The dotted lines denote the baseline value for the AUPRC, indicating the case of a random classifier.

Our first aim was to recover the emergence of IPF-specific alveolar macrophages overexpressing *SPP1* and other ECM-remodeling genes contributing to lung fibrosis^35^. Comparing different designs, the ACR design outperformed the AR and CR designs in detecting macrophages with the strongest profibrotic signature (Fig. 6b and Extended Data Fig. 7a). Interestingly, the CR design incorrectly prioritized neighborhoods with significantly fewer samples compared to true positives (Extended Data Fig. 7b,c), suggesting that the difference in ACR and CR design performance is due to residual batch effects in the latent space (Supplementary Fig. 3).

**Extended Data Fig. 8:**
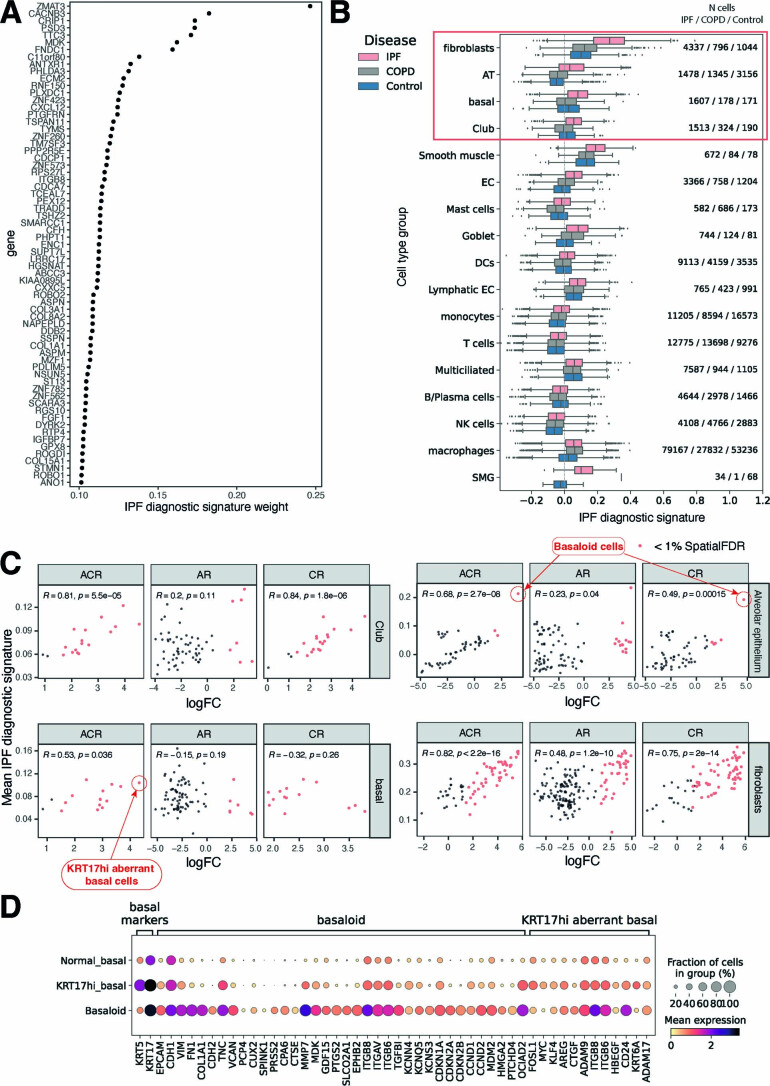
Detection of IPF diagnostic gene signature in stromal and epithelial lung cells. (**a**) Scatterplot of weights assigned to genes used for IPF signature calculation (from ref. ^36^). (**b**) Boxplots of IPF diagnostic signature values for cells of different cell type groups. Cells are grouped by disease status (Control: n = 28 patients; IPF: n = 32 patients). The number of cells for each cell type group and disease group is shown on the right. Cell type groups are ordered by the difference in mean signature between cells from IPF patients and COPD patients (COPD: chronic obstructive pulmonary disease), with cell type groups where the IPF diagnostic signature was highest in IPF patients shown on top. EC: endothelial cells; Club: club cells; SMG: submucosal gland cells. In boxplots the center line denotes the median; box limits, first and third quartiles; whiskers, 1.5X interquartile range. (**c**) Scatterplots of differential abundance log-Fold Change (DA logFC) against the mean expression of IPF diagnostic signature in cell neighbourhoods of affected cell type groups (AT: alveolar cells, basal cells, club cells, fibroblasts) with ACR design (left), AR design (middle) and CR design (right). Coloured points indicate neighbourhoods where the enrichment was significant (1% SpatialFDR and logFC > 0). Pearson’s correlation coefficients and p-values for significance of the correlation are reported (two-sided test). Neighbourhoods corresponding to aberrant basal-like phenotypes examined in downstream analysis are highlighted. (**d**) Dotplot of expression of marker genes for different aberrant basal-like cell states (KRT17hi aberrant basal markers from Jaeger et al.^40^, basaloid markers from Adams et al.^2^). Dot size is proportional to the fraction of cells expressing the gene in a group.

We next focused on stromal and epithelial cells. We considered cell types with high expression of biomarker genes from diagnostic models built on IPF lung explant RNA-seq^36^ (Extended Data Fig. 8a,b and Methods). The ACR design consistently led to the most precise distinction of cell states expressing the diagnostic signature (Fig. 6c and Extended Data Fig. 8c). Differential analysis using control samples led to the precise identification of rare aberrant cell states emerging in IPF, such as the *KRT5*–*KRT17*^+^ basaloid cells^2,37^ thought to originate from the alveolar epithelium in response to fibrosis^38,39^ (Extended Data Fig. 8c,d). Furthermore, the difference in performance between reference designs was especially notable for basal cells (Fig. 6c and Extended Data Fig. 8c). These were on average significantly enriched in the IPF samples, in agreement with previous reports^2,37^. However, by using the ACR design, we distinguished the neighborhoods of normal basal cells (with a mix of cells from patients with IPF and controls) and IPF-enriched neighborhoods with high biomarker expression (Extended Data Fig. 8c). We found that basal cells in the ACR design IPF-enriched neighborhoods overexpressed marker genes for *KRT5*^+^*KRT17*^hi^ aberrant basal cells identified in bronchial brushings of patients with IPF^40^ (Fig. 6d). Marker gene expression was especially high in the neighborhood showing the strongest enrichment in IPF cells. DA analysis with the CR or AR design did not distinguish this aberrant phenotype (Fig. 6d).

**Extended Data Fig. 9:**
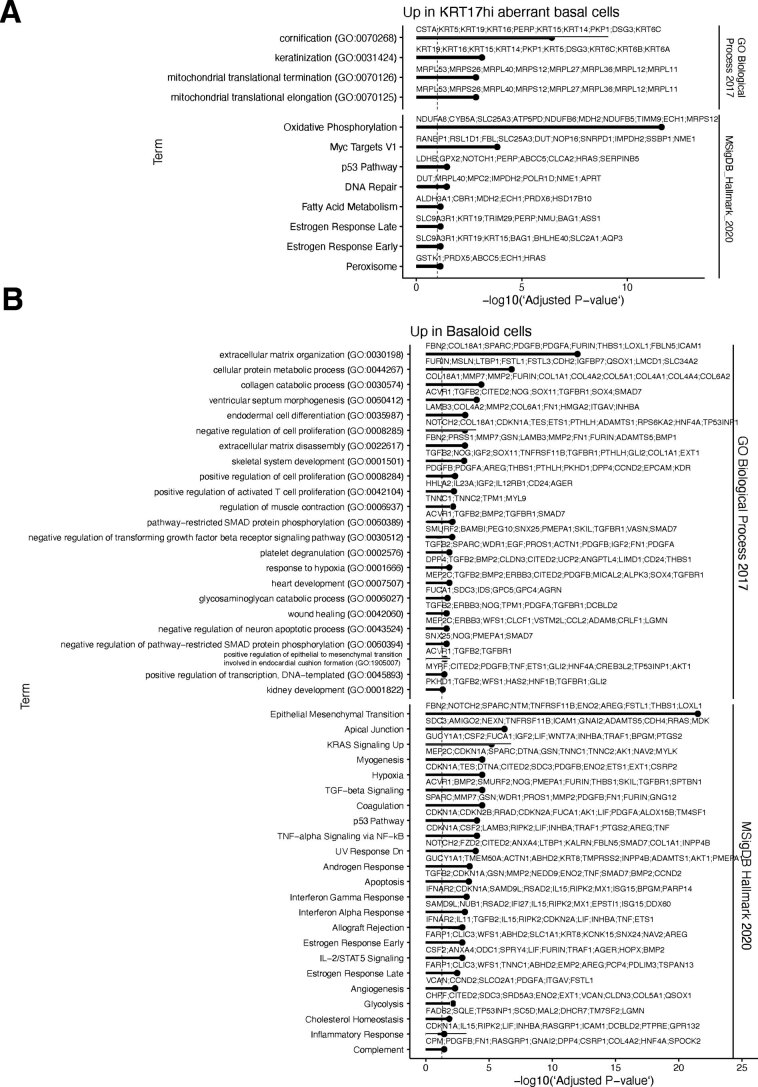
Differential expression analysis to identify markers for aberrant basal-like cells detected with ACR design. (**a**) Gene set enrichment analysis (Enrichr^80^ hypergeometric test) results for markers of KRT17hi aberrant basal cells: adjusted p-value (BH correction for multiple testing, transformed to - log10(p-val)) for significant gene sets (10% FDR threshold, marked by dotted line) from GO biological process terms and MSigDB Hallmark pathway terms. Example marker genes associated with each term are shown. (**b**) Gene set enrichment analysis (Enrichr^80^ hypergeometric test) results for markers of basaloid cells: adjusted p-value (BH correction for multiple testing, transformed to - log10(p-val)) for significant gene sets (5% FDR threshold, marked by dotted line) from GO biological process terms and MSigDB Hallmark pathway terms. Example marker genes associated with each term are shown.

While the study describing *KRT5*^+^*KRT17*^hi^ basal cells highlighted their transcriptional similarity to basaloid cells^40^, we identified both aberrant phenotypes as distinct states (Fig. 6e and Extended Data Fig. 8d). Therefore, we further characterized their specific markers and functional differences. Specifically, we identified genes differentially expressed between aberrant basal-like states and overexpressed compared to normal basal cells (Methods). We identified 981 significantly differentially expressed genes (DEGs) (FDR = 5%) (Fig. 6f and Supplementary Table 4), including six previously described markers for *KRT17*^hi^ aberrant basal cells and 35 previously described markers for basaloid cells. Several other previously described markers were only overexpressed compared to normal basal cells (Supplementary Fig. 4). *KRT17*^hi^ basal cells overexpressed genes associated with Myc signaling, in agreement with Jaeger et al.^40^, and genes involved in keratinization, including keratins and desmoplakin genes (Extended Data Fig. 9a). Similar processes have been identified in lung carcinoma^41^ and in the lung epithelium of smokers^42^, indicating that this might be a widespread response to epithelial injury. Basaloid-specific markers showed significant enrichment in the genes involved in ECM organization and epithelial–mesenchymal transition (EMT), including collagens and metalloproteases, as well as morphogenesis factors, including *SOX11*, *SOX4* and TGF-beta signaling genes (Extended Data Fig. 9b). These markers also include genes linked to genomic variants associated with lung function, including the EMT-inducer *IL32* (ref. ^43^), neurotrimin (*NTM*), *GPC5* and *DCBLD2* (refs. ^44–46^). Some of the newly identified markers encode targets of drugs approved or in trial for other lung pathologies. For example, *CSF2*, strongly overexpressed in basaloid cells, has been implicated in the pathogenesis for asthma and COPD, and is being investigated in phase 3 trials for pneumonia treatment (ClinicalTrials.gov registration: NCT04351152)^47^; the CCL2-inhibitor carlumab has completed a phase 2 trial for pulmonary fibrosis (ClinicalTrials.gov registration: NCT00786201); while U.S. Food and Drug Administration-approved drugs inhibiting *ROS1* are used for non-small cell lung carcinoma^48^.

## Discussion

In this study, we assessed how the choice of reference dataset affects the identification of altered cell states from scRNA-seq data of diseased tissues. Using simulations and real-life applications, we showed that atlas datasets are not a substitute for control samples, but that they enhance disease-state discovery in complex scenarios. Contrasting cell profiles from disease samples against a restricted set of control samples is necessary to minimize false positives in disease-state identification. However, when an atlas dataset is available, it is possible to reduce the number of control samples without introducing false discoveries and with minimal impact on sensitivity (Fig. 4a).

Multiple factors could explain the improved performance of ACR compared to CR design in complex scenarios. First, feature selection in joint embedding with a CR design is less likely to include disease-relevant genes necessary to distinguish rare populations (Fig. 3c). Additionally, residual batch effects in the latent space can lead to false positives (Extended Data Fig. 7b,c). Interestingly, while a comprehensive representation of cell states in atlas datasets might have a role, our leave-one-out analysis indicates that the size and composition of the atlas dataset do not significantly impact disease-state detection performance (Supplementary Note 2.2.1). Moreover, as in the comparison between tissue-specific or cross-tissue atlas datasets (Supplementary Note 2.2.2), sensitive detection of disease-specific states is possible when the cell type composition of atlas and case-control datasets differ substantially.

Despite its advantages, researchers may face challenges when applying an ACR design. First, data integration and harmonization efforts are ongoing, and integrated datasets are frequently updated with more individuals, even for well-sampled tissues, such as blood, lung^16^, heart^49–51^ or gastrointestinal tract^52,53^. Reassuringly, we showed that the ACR design is robust to the set of harmonized datasets (Supplementary Note 2.2.1) and maintains high sensitivity with smaller atlas datasets (Fig. 4b and Supplementary Fig. 2), making disease analysis more robust to atlas updates. Second, downloading and processing atlas data can be computationally expensive. By benchmarking disease-state detection using latent embedding with transfer learning^9^, we advocate for atlas builders to share trained models for embedding along with datasets (for example, refs. ^54–56^). Lastly, when the use of an atlas is not feasible, we found that in several benchmarking scenarios, a CR design with joint embedding provided satisfactory performance, serving as an alternative design in this scenario. In this case, we recommend validating predicted disease-associated states by checking for residual batch effects between samples (Extended Data Fig. 7b,c), and evaluating the robustness of results to factors such as the inclusion or exclusion of specific control samples (Fig. 4a) or feature selection (Fig. 3c).

Our disease cohort analyses revealed that an ACR design enables more sensitive identification of transitional and heterogeneous pathological cell states. In the COVID-19 dataset^11^, we captured IFN^hi^ states across immune cell types, and fine subsets of dysfunctional CD14^+^ monocytes associated with disease severity (Fig. 5e–g)^23^. Analyzing lung data from patients with IPF using an ACR design, we distinguished and characterized rare basal-like aberrant cell states (Fig. 6d–f). Previous studies linked IPF severity with basal marker gene expression^2,57–59^ and basal cell accumulation in distal airways^60^. Our analysis adds insights on basal-like cellular phenotypes in IPF. First, while *KRT17*^hi^ aberrant basal cells were first described in bronchial epithelium^40^, we found them in lung parenchyma, supporting their role in bronchiolization^61^. Second, we showed that only a subset of basal cells in the IPF samples were *KRT17*^hi^, suggesting that normal basal cells might undergo reprogramming in the parenchyma. Third, we established that *KRT17*^hi^ aberrant basal cells are distinct from the recently described IPF-associated *KRT5–KRT17*^+^ basaloid cells^2,37,62,63^, highlighting their distinguishing features and marker genes.

In conclusion, we demonstrated that the combined use of a cell atlas and matched controls as references enables the most precise identification of affected cell states in disease scRNA-seq datasets. We envision that our analysis will instruct the design of new cohort studies, guide efficient data reuse and provide operating principles for analysis of disease datasets and construction of cell atlases.

## Methods

### Ethics statement

This study relies on the analysis of previously published data, which were collected with written informed consent obtained from all participants and comply with the ethical guidelines for human samples.

### PBMC data preprocessing

We collected raw gene expression counts and cell type annotations from healthy PBMC 10X Genomics scRNA-seq data from 13 studies^5,18,22–24,30,54,64–69^, available via the CELLxGENE portal (https://cellxgene.cziscience.com/collections) (Supplementary Table 1). During harmonization, we sampled 500 cells for each sample to reduce the computational burden of this analysis, while maintaining sample-level diversity; we excluded samples for which fewer than 500 cells were detected, retaining in total 1,268 samples from 1,248 individuals. We subsequently filtered cells where at least 1,000 mRNA molecules were detected and genes that were expressed in at least one cell. This resulted in a dataset of 599,379 high-quality cells.

To generate a unified cell type annotation, we integrated all normal cells from different studies in a common latent space using the scVI model, as implemented in the Python package scvi-tools^26,70^. Briefly, we selected the 5,000 most HVGs based on the dispersion of log-normalized counts, as implemented in SCANPY^71^. We trained the scVI model on raw counts, subsetting to HVGs, considering the library ID as batch (model parameters: *n*_latent = 30, gene_likelihood = ‘nb’, use_layer_norm = ‘both’, use_batch_norm = ‘none’, encode_covariates = True, dropout_rate = 0.2, *n*_layers = 2; training parameters: early_stopping = True,train_size = 0.9, early_stopping_patience = 45, max_epochs = 200, batch_size = 1,024,limit_train_batches = 20). We constructed a *k*-nearest neighbor graph based on similarity in the scVI latent dimensions, using *k* = 50. Cells were clustered using the Leiden algorithm with resolution = 1.5. Subsequently, clusters were annotated by majority voting using the harmonized cell type labels available via CELLxGENE. During this process, one cluster of cells was excluded as potentially containing doublets. After this final filtering, the dataset included 597,321 cells annotated into 16 cell types.

### Simulation experiments

In this section we describe the simulation strategy (Fig. 2a) and workflow to identify OOR cells (Fig. 2b). We designed evaluation experiments and chose methods for the integration and differential analysis with the specific use-case of disease datasets in mind. We believe our results will extrapolate to other types of case-control studies, as long as the main assumptions apply, that is, (1) that all the cell states observed in the control dataset are also found in the atlas dataset and (2) that only a fraction of cell types are altered in the disease datasets. Note that throughout this study the term ‘cell state’ defines a group of cells that are more transcriptionally similar to each other than to other cells in the same tissue.

### Data splitting into atlas, control and pseudo-disease

To simulate the attributes of the disease, atlas and control datasets, we selected donors from one study (query study, 29 healthy donors, Stephenson et al.^24^) and we split these at random with equal probabilities into a disease subset (16 donors) and a control subset (13 donors). The data from the remaining 12 studies comprises the atlas dataset (1,219 donors). To simulate the presence of an OOR cell state, we selected one cell type label and removed all cells with that label from the control and atlas dataset. We repeated this simulation with 15 annotated cell types in the PBMC dataset. Neutrophils were excluded because they were underrepresented in the Stephenson et al.^24^ study. For seven cell types where the number of cells in the OOR cell state was fewer than 250 cells, we found that our workflow was unable to detect OOR states across designs (Extended Data Fig. 4); therefore, most downstream analysis was restricted to simulations where at least 250 OOR cells were simulated.

To simulate a scenario with multiple cell states altered in disease with different effect sizes (Fig. 3d–f), we selected a fixed cell type label to be removed from the atlas and control as described above (classical monocytes). We then selected a variable cell type label (shifted OOR cell state) that we split between an OOR and an in-reference group with the following procedure: we selected the cells of the shifted OOR cell state in the disease and control datasets; we log-normalized their gene expression profiles and ran a PCA to split the cells into OOR and in-reference groups based on their weights on the first principal component. We then used a *k*-nearest neighbor classifier (using the implementation in scikit-learn, with *k* = 10) to assign atlas cells to one of the two groups. We used this procedure instead of running the PCA on atlas, control and disease cells to avoid having a first principal component that captures only batch effects between the query and atlas datasets.

### Latent space embedding

For each simulated atlas, control and disease dataset assignment, we embedded the reference and query datasets into a common latent space using transfer learning with scArches^9^ on scVI models^9,26^, using the implementation in the Python package scvi-tools v.0.17.4 (ref. ^70^). Briefly, we selected the 5,000 most HVGs in the reference dataset based on the dispersion of log-normalized counts, as implemented in SCANPY. We trained the scVI model on the raw counts of the reference dataset, subsetting to HVGs, considering the sample ID as batch and specifying the recommended parameters to enable scArches mapping (use_layer_norm = ‘both’, use_batch_norm = ‘none’, encode_covariates = True, dropout_rate = 0.2, *n*_layers = 2). Models were trained for 400 epochs or until convergence. For the CR design with joint embedding (CR scVI), the scVI model was trained on the concatenated disease and control datasets. Next, we performed transfer learning on the query dataset(s) from the model trained on the reference, running the model for 200 epochs and setting the weight_decay parameter to 0. The reference (for scVI training) and query (for scArches mapping) datasets for latent space embedding were defined as follows for the three reference designs: AR design: the atlas dataset was used as the reference dataset, the disease dataset was used as the query dataset; control reference with query mapping (CR design, scArches): the control dataset was used as the reference dataset, the disease dataset was used as the query dataset; control reference with joint embedding (CR design, scVI): the control and disease datasets were used as the reference dataset, no query mapping was performed; ACR design: the atlas dataset was used as the reference dataset, the disease and control datasets were used as the query dataset.

### DA analysis

To find cell states enriched in the disease dataset, we used the Milo framework for DA on cell neighborhoods^11^ using the implementation in the package milopy v.0.1.0 (https://github.com/emdann/milopy). Briefly, we computed the *k*-nearest neighbor graph of cells in the reference and disease datasets based on latent embedding. The reference datasets for differential analysis were defined as follows for the three reference designs: (1) AR design: atlas dataset; (2) CR design: control dataset; (3) ACR design: control dataset.

Of note, for the ACR design, the atlas dataset was not considered when constructing the *k*-nearest neighbor graph. This reduces the computational burden of handling a dataset of hundreds of thousands of cells. We set the value of *k* to be equal to the total number of samples times five, up to a maximum of *k* = 200 (this upper limit was set for memory efficiency reasons), as suggested by Dann et al.^11^. We assigned cells to neighborhoods (milopy.core.make_nhoods, parameters: prop = 0.1) and counted the number of cells belonging to each sample in each neighborhood (milopy.core.count_cells). We assigned to each neighborhood a cell type label based on majority voting of the cells belonging to that neighborhood. To test for enrichment of cells from the disease dataset, we modeled the cell count in neighborhoods as a negative binomial generalized linear model, using a log-linear model to model the effects of disease status on cell counts (log fold change). Although the split between control and disease samples was balanced in terms of the available metadata, in the query study there was a known batch effect between the three sites from which samples were collected^24^. Therefore, we included site identity as a confounding covariate in the DA model when using the ACR and CR designs, although we found that the results presented in this report were robust even without modeling this confounder. We controlled for multiple testing using the weighted Benjamini–Hochberg correction as described in Dann et al.^11^ (spatial FDR correction). Unless otherwise specified, neighborhoods were considered enriched in disease cells if the spatial FDR < 0.1 and log fold change > 0.

For the comparison across DA methods (Extended Data Fig. 3), we constructed the *k*-nearest neighbor graph using the same parameters as described above for the Milo analysis. We used the MELD^13^ implementation available via PypI (v.1.0.0) and tested for significant differences in density between pseudo-disease and control samples as described by Petukhov et al.^72^. Specifically, we computed sample-specific densities over the *k*-nearest neighbor graph (running meld.MELD().fit_transform()) and tested for significant differences in sample densities between conditions using a Wilcoxon rank-sum test, as implemented in SciPy^73^. While in the original MELD analysis the authors took the normalized mean density across samples of the same condition as a metric for the effect size of DA, we opted to use the Wilcoxon rank-sum test after observing significant variance in sample densities across donors of the same condition. We ran covarying neighborhood analysis (CNA)^15^ using the implementation available via PypI (v.0.1.4). We used the CNA correlation as a metric for the effect size of DA (running cna.tl.association, with *ks* = [20]).

We tested additional alternatives to DA to identify OOR cell states, as shown in Supplementary Note 2.1.

### Sensitivity analysis

For each simulation (that is, with different OOR cell state and reference design), we defined a neighborhood as an OOR state (true positive) if the percentage of OOR cells in the neighborhood was more than 20% of the maximum percentage observed in that simulation. This threshold selection aimed to quantify the ability to detect the neighborhoods where the largest number of OOR cells was found, even when the atlas dataset was included in the *k*-nearest neighbor graph (AR design); most cells in the neighborhoods always belong to the atlas dataset. The selected thresholds for each experiment are shown in Extended Data Fig. 1. We calculated TPRs, FPRs and FDRs considering neighborhoods where the spatial FDR < 0.1 and log fold change > 0 as predicted positives.

With precision-recall curve analysis, we quantified the ability to detect true positive OOR states with different thresholds of log fold change, without considering the significance estimated with spatial FDR, using the implementation in scikit-learn^74^. As a measure of uncertainty around the estimated AUPRC, we performed bootstrap resampling on the neighborhood log fold change values, maintaining the original ratio of positive and negative points, and computed the 95% CI on the distribution of AUPRC values for 1,000 resamplings.

### Control and atlas size analysis

For the analysis with varying number of control donors (Fig. 4a and Supplementary Fig. 1), we selected the simulations with the five OOR cell populations with the highest average TPR with CR and ACR designs in the previous analysis (Fig. 3b). For each simulation, we selected the five, seven or nine donors from the disease dataset who had the highest fraction of cells in the OOR cell population. Subsequently, we selected a random subset of *n* donors (with 3 < *n* < 12) from the control dataset and performed disease-state identification with the CR or ACR design, as described above. For each disease dataset size and $n$ we repeated the simulation with five different initializations of the control donor selection.

To assess whether a shallow atlas dataset would introduce false discoveries in disease-state identification (Supplementary Fig. 2), we used all 29 donors from the query dataset in the disease and control datasets, and subsampled the atlas dataset removing data from one to 11 studies (ordering studies according to the total number of cells), and performed disease-state identification with the AR and ACR designs.

More cases of robustness to perturbation of the atlas and control datasets of the reference designs are described in Supplementary Notes 2.1 and 2.2.

### Design comparison on the COVID dataset

#### Data preprocessing and model training

We downloaded data for COVID-19 and healthy PBMCs from Stephenson et al.^24^, via the CELLxGENE portal (collection ID: ddfad306-714d-4cc0-9985-d9072820c530). We sampled 500 cells for each sample to reduce the computational burden of this analysis, while maintaining sample-level diversity, and we excluded samples for which fewer than 500 cells were detected. We excluded cells where fewer than 1,000 mRNA molecules were detected and we excluded data from three samples that were profiled with the Smart-seq2 protocol. As cell type annotation, we used the high-level annotation from the original authors.

As the atlas dataset, we used the healthy PBMC data described above, excluding the healthy PBMC profiles from Stephenson et al.^24^. Reference model training and query mapping was performed as described above. After query mapping, control and COVID-19 cells were embedded in a *k*-nearest neighbor graph (*k* = 100), which was used to build neighborhoods and perform DA with Milo as described above. For the comparison of de novo integration and query mapping (Fig. 5d), scVI training was performed on the concatenated atlas, control and COVID-19 datasets (ACR design) or control and COVID-19 datasets (CR design), as described above. Also in this case, the atlas dataset was used for scVI model training, but only model weights were used for mapping with scArches; all downstream analysis was performed solely on the COVID-19 and control datasets.

#### IFN signature calculation

To define IFN-stimulated cells, we aggregated the expression of a set of IFN-associated genes defined by Yoshida et al.^22^. (*BST2*, *CMPK2*, *EIF2AK2*, *EPSTI1*, *HERC5*, *IFI35*, *IFI44L*, *IFI6*, *IFIT3*, *ISG15*, *LY6E*, *MX1*, *MX2*, *OAS1*, *OAS2*, *PARP9*, *PLSCR1*, *SAMD9*, *SAMD9L*, *SP110*, *STAT1*, *TRIM22*, *UBE2L6*, *XAF1* and *IRF7*), using the SCANPY function scanpy.tl.score_genes() to quantify signature expression for each cell. The signature was calculated as the average scaled expression of the IFN-associated genes, which was subtracted from the average expression of a reference set of genes sampled for each binned expression value^75^. A threshold of IFN signature greater than 0.05 was used for the precision-recall analysis.

#### CD14^+^ monocyte disease-state analysis

For the analysis of the COVID-19-associated monocyte subsets, we focused on the neighborhoods annotated as CD14^+^ monocytes based on majority voting, as described above. We split CD14^+^ monocyte neighborhoods into IFN^hi^ COVID-19 neighborhoods (spatial FDR < 0.1, log fold change > 0 and IFN signature > 0.2), IFN^lo^ COVID-19 neighborhoods (spatial FDR < 0.1, log fold change > 0 and IFN signature < 0.2) and healthy neighborhoods (the remaining neighborhoods). To assign cells to one of these three phenotypes, we computed, for each cell, the number of neighborhoods of each phenotype to which that cell belonged (as Milo neighborhoods can be partially overlapping) and we labeled cells based on the most representative phenotype (if the cell was found in at least three neighborhoods of that phenotype; otherwise the cell was annotated as mixed CD14^+^ monocyte phenotype).

For differential expression analysis, we aggregated gene expression profiles by summing counts according to sample and CD14^+^ monocyte phenotype and performed differential expression testing with the edgeR quasi-likelihood test^76^ using the implementation in the R package glmGamPoi^76^ and 1% FDR (Supplementary Tables 2 and 3).

### Design comparison on the IPF dataset

#### Data preprocessing and model training

Gene expression count matrixes for human lung IPF, control and COPD scRNA-seq data from Adams et al.^2^ were downloaded from the Gene Expression Omnibus (accession no. GSE136831). As cell type annotations, we used uniform labels generated from the integration of this dataset with the HLCA by Sikkema et al.^16^, downloaded from *Zenodo* (https://zenodo.org/record/6337966). For latent embedding with the AR and ACR designs, we used the embeddings from scArches mapping on the core HLCA model provided by Sikkema et al. via *Zenodo*. For latent embedding with the CR design, we trained a scANVI model^77^ on the concatenated control and disease replicating the parameters used to train the HLCA model (according to the notebooks in https://github.com/LungCellAtlas/HLCA_reproducibility), using dataset ID as the batch covariate and training on the same set of 2,000 HVGs used for HLCA training. We opted to keep the HLCA HVG set for the CR design instead of recomputing HVGs because it was selected using a custom batch-aware strategy and compared (in the original study) to alternative selections with a benchmarking pipeline^16^. Therefore, we reasoned that recomputing HVGs on the CR design would not represent a fair comparison. DA with Milo was performed as described above (changing only milopy.core.make_nhoods, parameters: prop = 0.01), comparing the abundance of cells from IPF samples to the abundance of cells from the control samples. Neighborhood-level annotations were performed using majority voting as described previously.

#### *SPP1*^hi^ macrophage analysis

To define *SPP1*^hi^ profibrotic macrophages, we aggregated the expression of a set of marker genes defined by Adams et al.^2^ (*SPP1*, *LIPA*, *LPL*, *FDX1*, *SPARC*, *MATK*, *GPC4*, *PALLD*, *MMP7*, *MMP9*, *CHIT1*, *CSTK*, *CHI3L1*, *CSF1*, *FCMR*, *TIMP3*, *COL22A1*, *SIGLEC15*, *CCL2*), using the SCANPY function scanpy.tl.score_genes() to quantify the signature expression of each cell. A threshold of signature greater than 0.32 was used for the precision-recall analysis (corresponding to the 90% quantile of the signature expression in all cells). For comparison to the label transfer uncertainty metrics, we used the values for uncertainty provided by Sikkema et al.

#### IPF signature analysis

To define profibrotic signatures in stromal cells, we used a gene expression signature developed on bulk RNA-seq data to diagnose IPF from lung explants^36^. We downloaded DEGs from the original paper, selected upregulated genes and normalized the differential expression test effect sizes to weights $\in [\mathrm{0,1}]$ with L2 normalization (Extended Data Fig. 8a). We then used a modified version of the SCANPY function scanpy.tl.score_genes() (using weighted means based on gene weights) to quantify the diagnostic signature expression for each cell. We then selected relevant cell types where the difference in mean signature expression between cells from IPF samples and cells from COPD samples was the highest, to control for the effect of end-stage lung disease (Extended Data Fig. 8b). For the precision-recall analysis, we computed the mean profibrotic signature expression across IPF cells in the neighborhoods and used the top 50% quantile for each cell type group (alveolar type (AT), fibroblasts, club cells, basal cells) as the threshold for calling true positives.

#### Analysis of aberrant basal-like cells

We annotated the neighborhoods of basaloid cells and *KRT17*^hi^ aberrant basal cells based on profibrotic signature expression and expression of marker genes reported by refs. ^2,37,38,40^ (Extended Data Fig. 8a,c,d). We defined normal basal cells as cells annotated as basal and not belonging to the basaloid neighborhood or the *KRT17*^hi^ basal neighborhood. In total we annotated 1,562 normal basal cells, 377 basaloid cells and 350 *KRT17*^hi^ aberrant basal cells, distributed across individuals (Fig. 6e). For differential expression analysis, we aggregated gene expression profiles by summing counts according to sample and basal-like phenotype, and performed differential expression testing with the edgeR quasi-likelihood test (Robinson and Oshlack^78^) using the implementation in the R package glmGamPoi (Ahlmann-Eltze and Huber^76^), using 1% FDR (Supplementary Table 4). We compared *KRT17*^hi^ aberrant basal cells against basaloid cells, and each aberrant state against normal basal cells. Differential expression analysis was run on the top 7,500 most HVGs for each comparison, using the modelGeneVar function from the scran package^79^. We considered genes to be aberrant state markers (shown in Fig. 6f and Supplementary Fig. 4) only if significant in the comparison between aberrant states and significantly overexpressed against the normal state (reported in Supplementary Table 4). We performed gene set enrichment analysis using the enrichr method^80^ with implementation carried out using the Python package GSEApy^81^. To annotate genes targeted by drugs in trials or approved for lung disease, we downloaded the targets of drugs approved or being trialed for lung disease (trait ID: EFO_0003818) in the Open Targets platform^82^. To annotate genes associated with GWAS variants for lung function (forced expiratory volume, trait ID EFO_0004314), we downloaded a list of significant GWAS loci and predicted causal genes based on the locus2gene model available via the Open Targets Genetics database^83^. The full tables for drug targets, the lung function GWAS studies used for the genetic evidence analysis and GWAS-associated genes are shared as metadata in our reproducibility repository (https://github.com/MarioniLab/oor_design_reproducibility).

### Statistics and reproducibility

No statistical method was used to predetermine sample size. No data were excluded from the analyses, unless otherwise stated in the relevant section of the Methods where the rationale for exclusion is described. Statistical tests were chosen to model the underlying data distributions (negative binomial likelihood generalized linear models for cell counts^11^ and mRNA counts^78^, Wilcoxon signed-rank tests for nonparametric comparisons between metrics). The experiments were not randomized. The investigators were not blinded to allocation during the experiments and outcome assessments. All code to replicate the analysis is available as part of code availability.

### Reporting summary

Further information on research design is available in the Nature Portfolio Reporting Summary linked to this article.

## Online content

Any methods, additional references, Nature Portfolio reporting summaries, source data, extended data, supplementary information, acknowledgements, peer review information; details of author contributions and competing interests; and statements of data and code availability are available at 10.1038/s41588-023-01523-7.

### Supplementary information

Supplementary Information Supplementary Figs. 1–4, Notes, Note Figs. 1–6 and Note references.

Reporting Summary

Supplementary Table 1 References of studies included in the healthy PBMC dataset used for the simulations.

Supplementary Table 2 Differential expression analysis results for comparison of CD14^+^ monocyte COVID-19 phenotypes with ACR design. Differential expression analysis was performed using the edgeR quasi-likelihood test^64^ using the implementation in the R package glmGamPoi^65^, using 1% Benjamini–Hochberg FDR as the threshold for significance.

Supplementary Table 3 Differential expression analysis results for comparison of CD14^+^ monocyte COVID-19 phenotypes with CR design. Differential expression analysis was performed using the edgeR quasi-likelihood test^64^ using the implementation in the R package glmGamPoi^65^, using 1% Benjamini–Hochberg FDR as the threshold for significance.

Supplementary Table 4 Differential expression analysis results for comparison of Basaloid and KRT17hi aberrant basal cells in the IPF dataset. Differential expression analysis was performed using the edgeR quasi-likelihood test^65^ using the implementation in the R package glmGamPoi^65^, using 1% Benjamini–Hochberg FDR as the threshold for significance.

## Data Availability

All the data used for analysis are publicly available. The blood datasets used for the simulation studies and COVID-19 analysis were downloaded from the CELLxGENE database (Supplementary Table 1). Lung data from the IPF cohort are available via the Gene Expression Omnibus (accession no. GSE136831). The core Human Lung Cell Atlas gene expression data were downloaded from CELLxGENE database (ID 6f6d381a-7701-4781-935c-db10d30de293). Unified cell type annotations for healthy and IPF data were downloaded from Zenodo (https://zenodo.org/record/6337966). The Tabula Sapiens data used in Supplementary Note 2.2.2 were downloaded from figshare (https://figshare.com/articles/dataset/Tabula_Sapiens_release_1_0/14267219). All processed data objects in AnnData format^84^ and trained scVI models are available via figshare (10.6084/m9.figshare.21456645).

## Code availability

The functions for benchmarking out-of-reference state detection, including the code for preprocessing, data splitting, latent embedding, differential analysis and evaluation metrics, have been made available as a Python package at https://github.com/MarioniLab/oor_benchmark (deposited at Zenodo^85^). Notebooks and scripts to reproduce all analyses presented in the manuscript are available at https://github.com/MarioniLab/oor_design_reproducibility (deposited at Zenodo^86^).
