# Supplementary material for: Precise identification of cell states altered in disease using healthy single-cell references
Source: Nat Genet. 2023 Oct 12;55(11):1998–2008. doi: 10.1038/s41588-023-01523-7 (PMC10632138; doi:10.1038/s41588-023-01523-7)
Supplement: Supplementary file 1 — Supplementary Figs. 1–4, Notes, Note Figs. 1–6 and Note references. [file 41588_2023_1523_MOESM1_ESM.pdf]

nature genetics

Analysis

https://doi.org/10.1038/s41588-023-01523-7

Precise identification of cell states altered in disease using healthy single-cell references

In the format provided by the authors and unedited

Supplementary Information - Precise identification of cell states altered in disease using healthy single-cell references

Table of contents

Supplementary Information - Precise identification of cell states altered in disease using healthy single-cell references **1**
Table of contents 1
1. Supplementary Figures **2**
2. Supplementary Notes **10**
2.1 Alternatives to differential abundance analysis for OOR state detection. **10**
2.1.1 Distance/segregation of OOR cells in latent space 10
2.1.2 Mapping quality control metrics 10
2.1.3 OOR detection with label transfer and differential expression analysis 11
2.2 Robustness of OOR detection with ACR design **12**
2.2.1 Leave-one-out analysis 12
2.2.2 OOR state detection with cross-tissue atlas **13**
2.3 Matched controls allow the most sensitive out-of-reference detection **14**
2.4 Impact of latent embedding strategy on OOR state detection **15**
3. Supplementary Note Figures **17**
Supplementary Note References **26**

1. Supplementary Figures

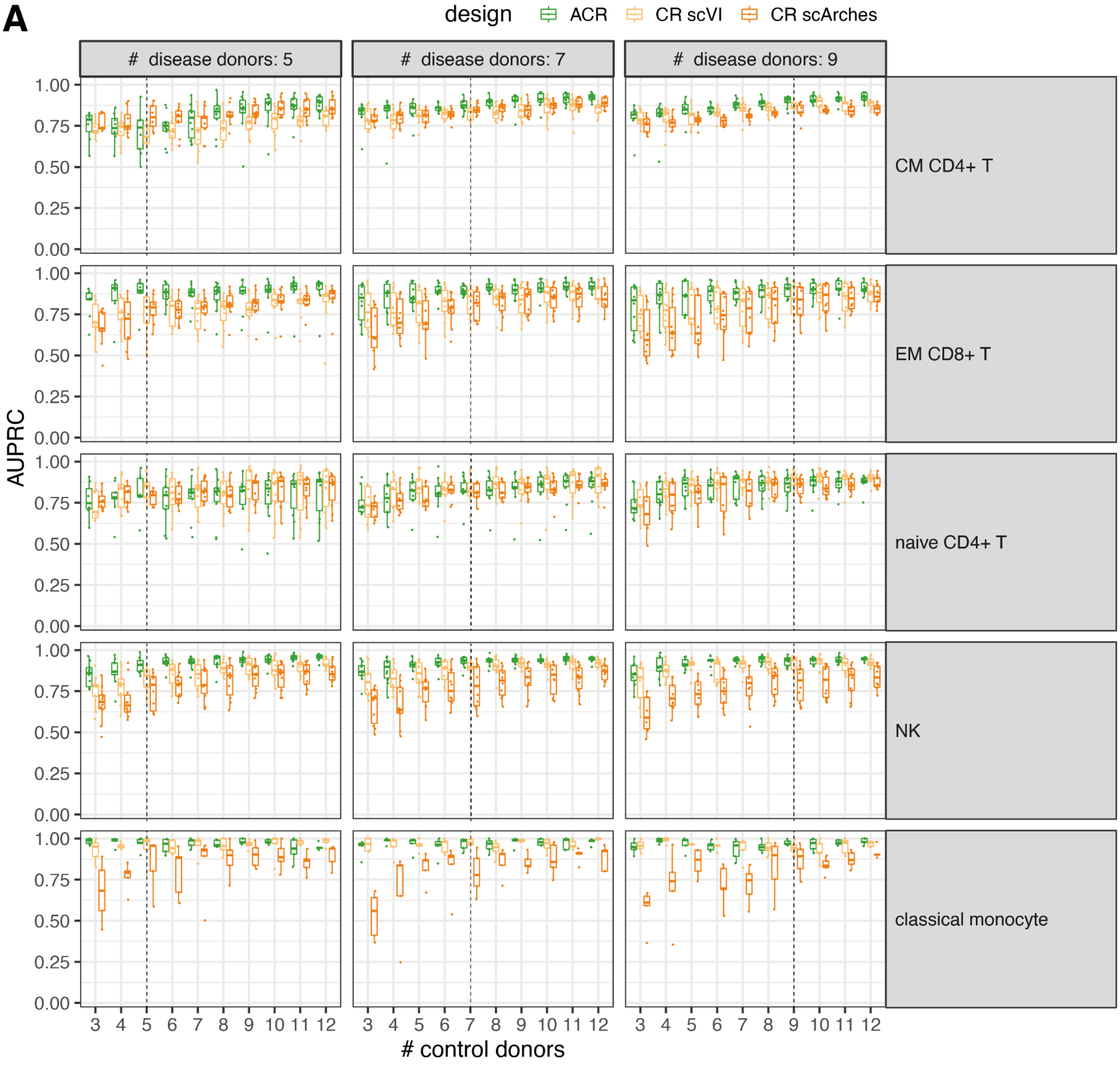
A
design
ACR
CR scVI
CR scArches
disease donors: 5
disease donors: 7
disease donors: 9
CM CD4+ T
EM CD8+ T
naive CD4+ T
NK
classical monocyte
AUPRC
1.00
0.75
0.50
0.25
0.00
3 4 5 6 7 8 9 10 11 12
control donors

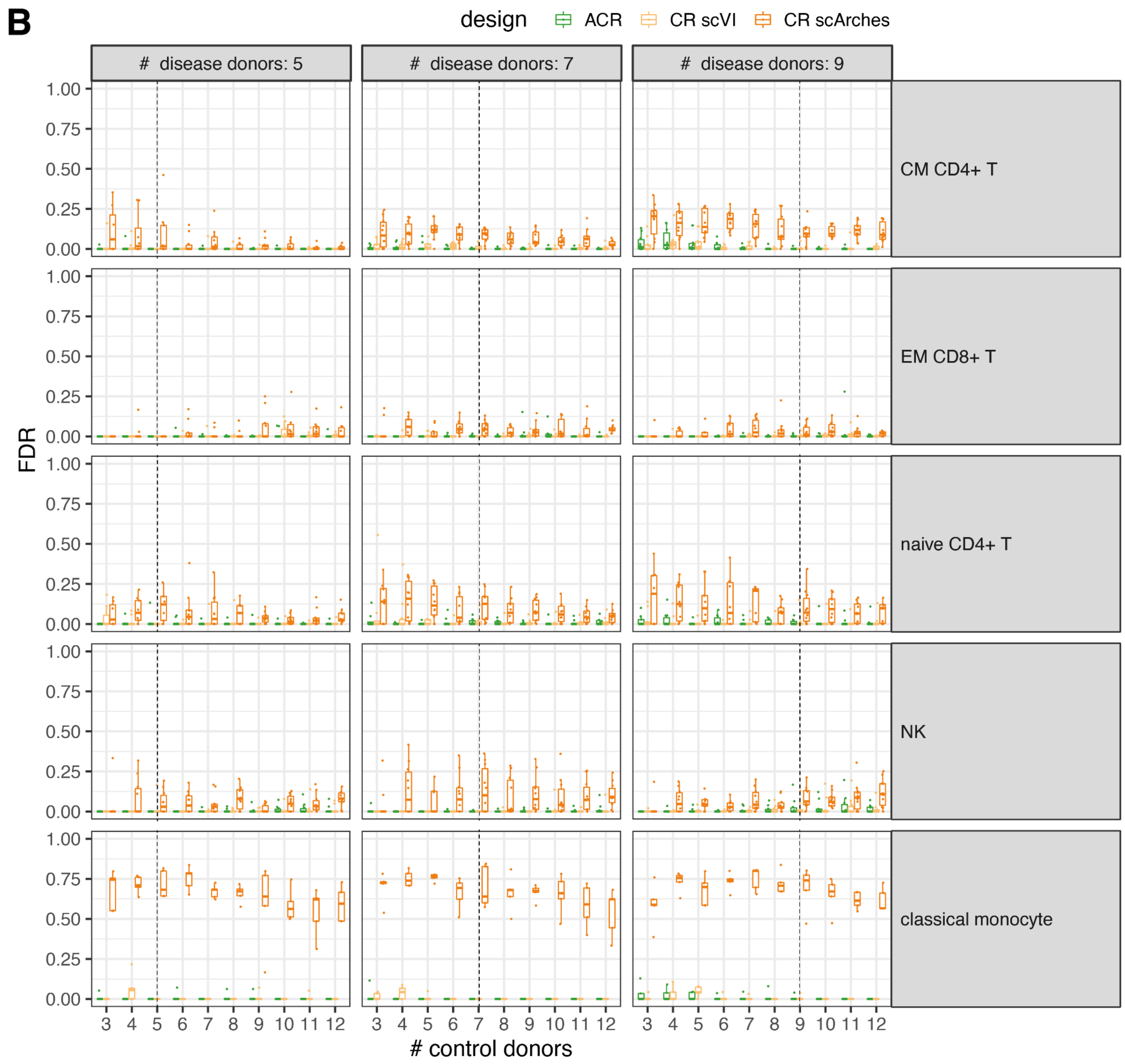
B
design
ACR
CR scVI
CR scArches
disease donors: 5
disease donors: 7
disease donors: 9
CM CD4+ T
EM CD8+ T
naive CD4+ T
NK
classical monocyte
FDR
1.00
0.75
0.50
0.25
0.00
3 4 5 6 7 8 9 10 11 12
control donors

C

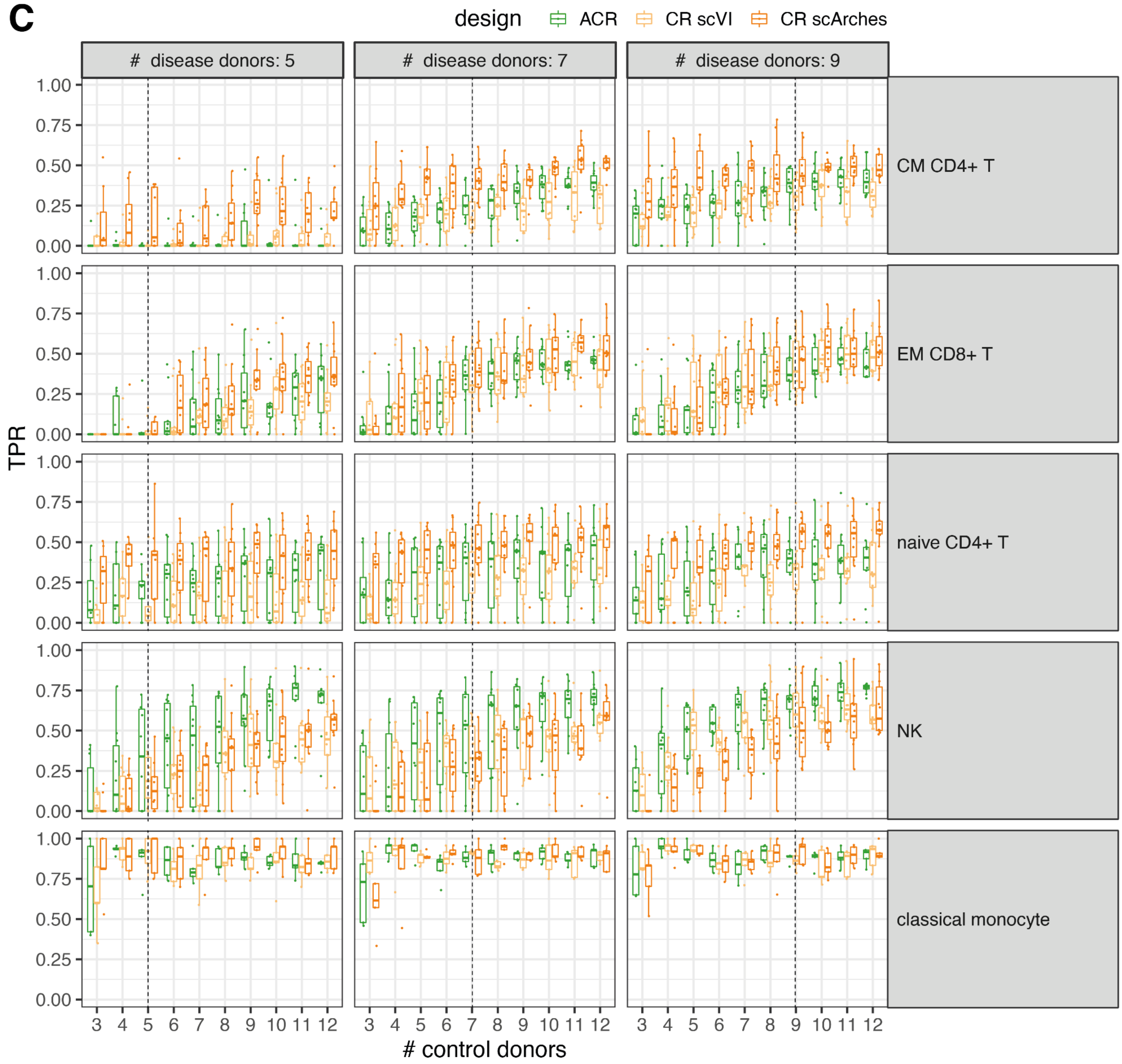

Supplementary Figure 1: ***Robustness of ACR and CR design to size of control cohort.*** *Boxplots of area under the precision-recall curve (AUPRC) (**A**), false discovery rate (FDR) (**B**) and true positive rate (TPR) (**C**) for simulations with increasing number of donors in the control dataset (x-axis), using atlas to control reference design (ACR, green) or control reference design with query mapping (CR scArches, orange) or joint embedding (CR scVI, yellow). Results from simulations with five different OOR cell states (y-axis facets), selected by top mean TPR across designs in* figure 3B, *and using 5 different samples of donors for each number of control donors and OOR state are shown. In these simulations 5, 7 or 9 donors were used in the disease dataset (x-axis facets), as indicated by the dotted line. In boxplots the center line denotes the median; box limits, first and third quartiles; whiskers, 1.5X interquartile range.*

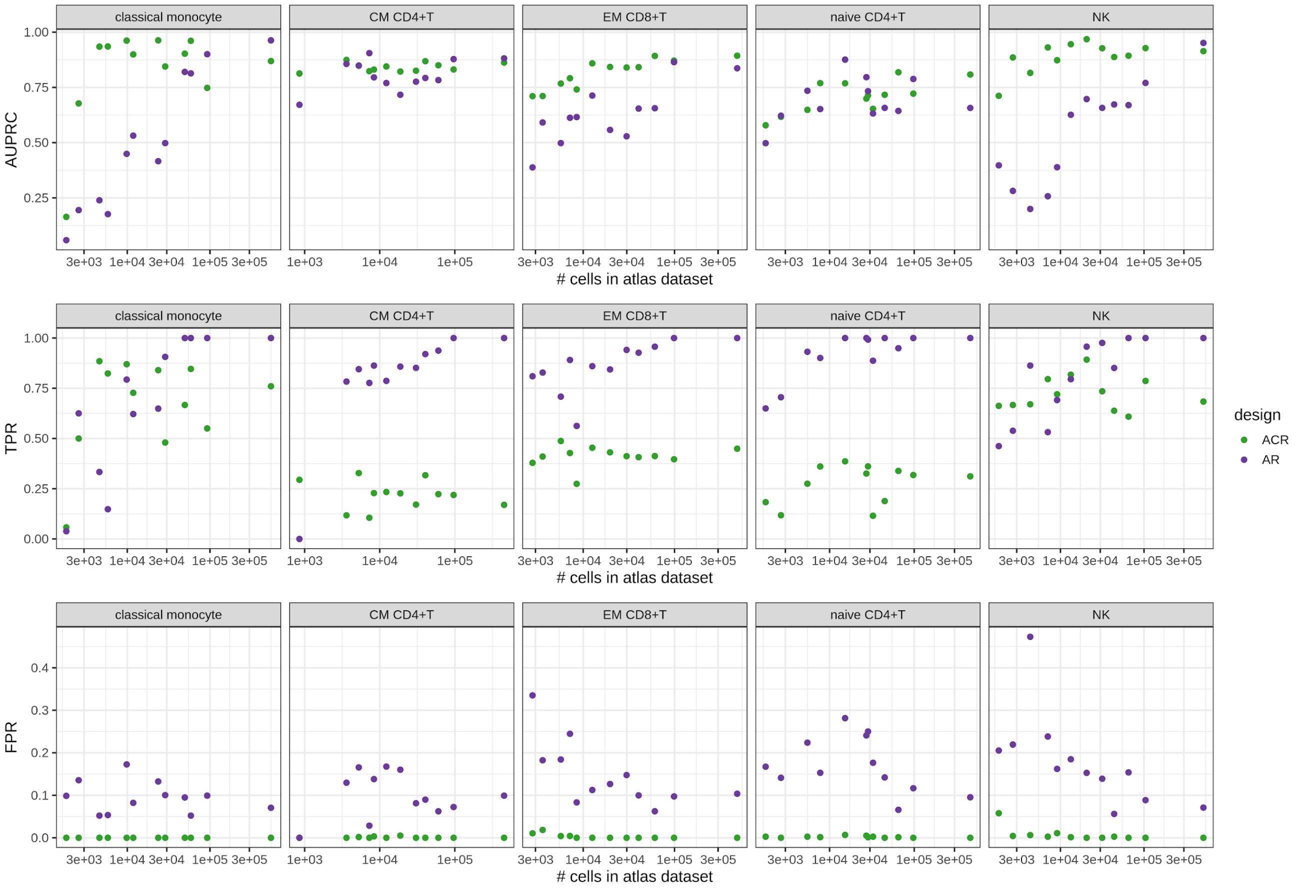

Supplementary figure ***2: True positives and false positives with decreasing size of atlas dataset.*** *Area under the precision-recall curve (AUPRC, top y-axis), true positive rate (TPR, middle y-axis) and false positive rate (FPR, bottom y-axis) of OOR state detection for simulations with an increasing number of studies and cells (x-axis) included in the atlas reference for AR and ACR design (colour). Results from simulations with five different OOR cell states are shown, selected by top mean TPR across designs in (*figure 3*B).*

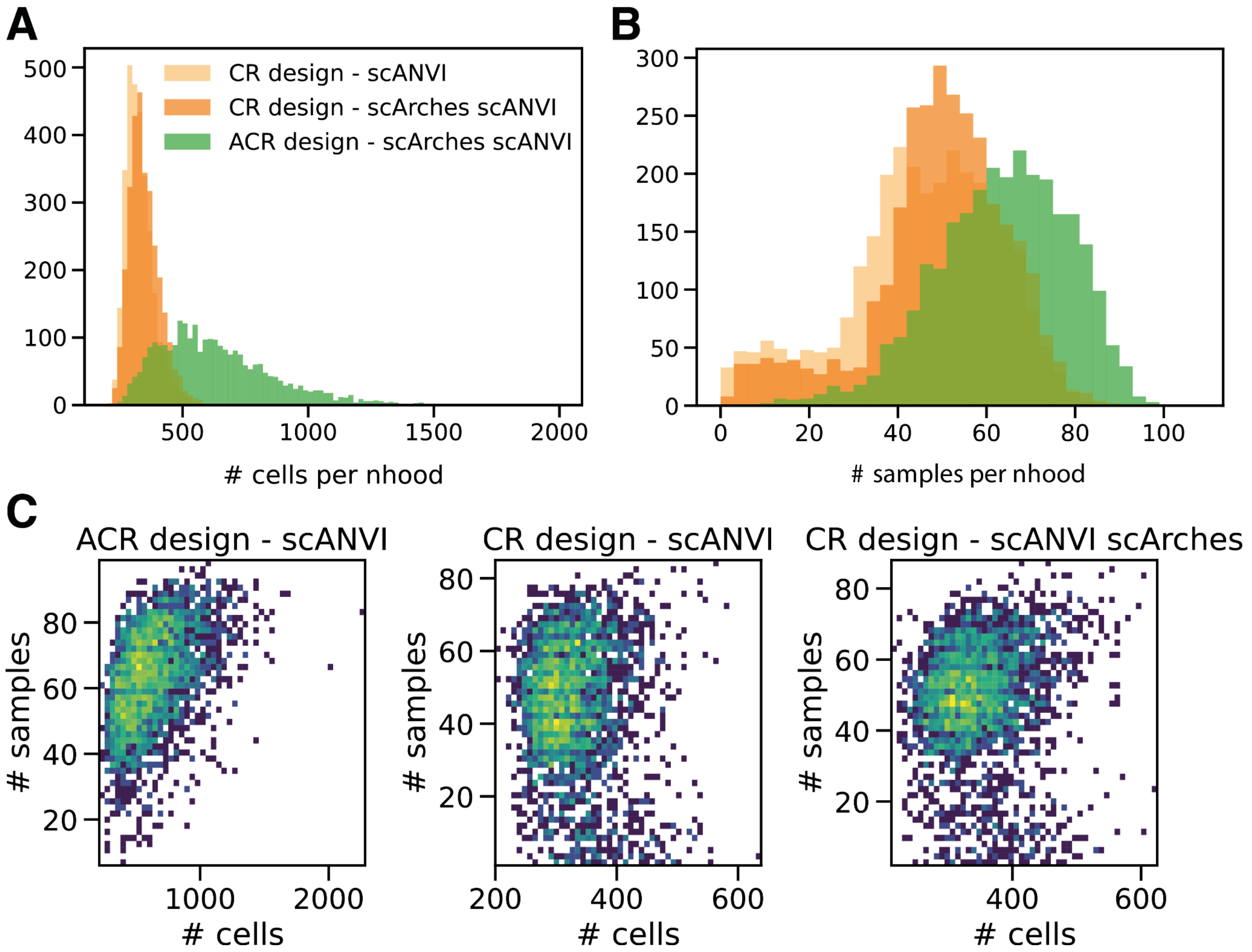

Supplementary figure 3: Cell and sample representation in cell neighbourhoods constructed with different reference designs on IPF case-control dataset. *Integration with CR design leads to less mixing between samples in the cell neighbourhoods (**A**) Histogram of number of cells per neighbourhood with query-mapping CR design (scANVI scArches), with joint embedding CR design (scANVI) and query mapping ACR design. (**B**) Histogram of number of samples per neighbourhood with query-mapping CR design (scANVI scArches), with joint embedding CR design (scANVI) and query mapping ACR design. (**C**) Two-dimensional histograms showing the number of cells in neighbourhood against the number of samples in neighbourhood for each reference design.*

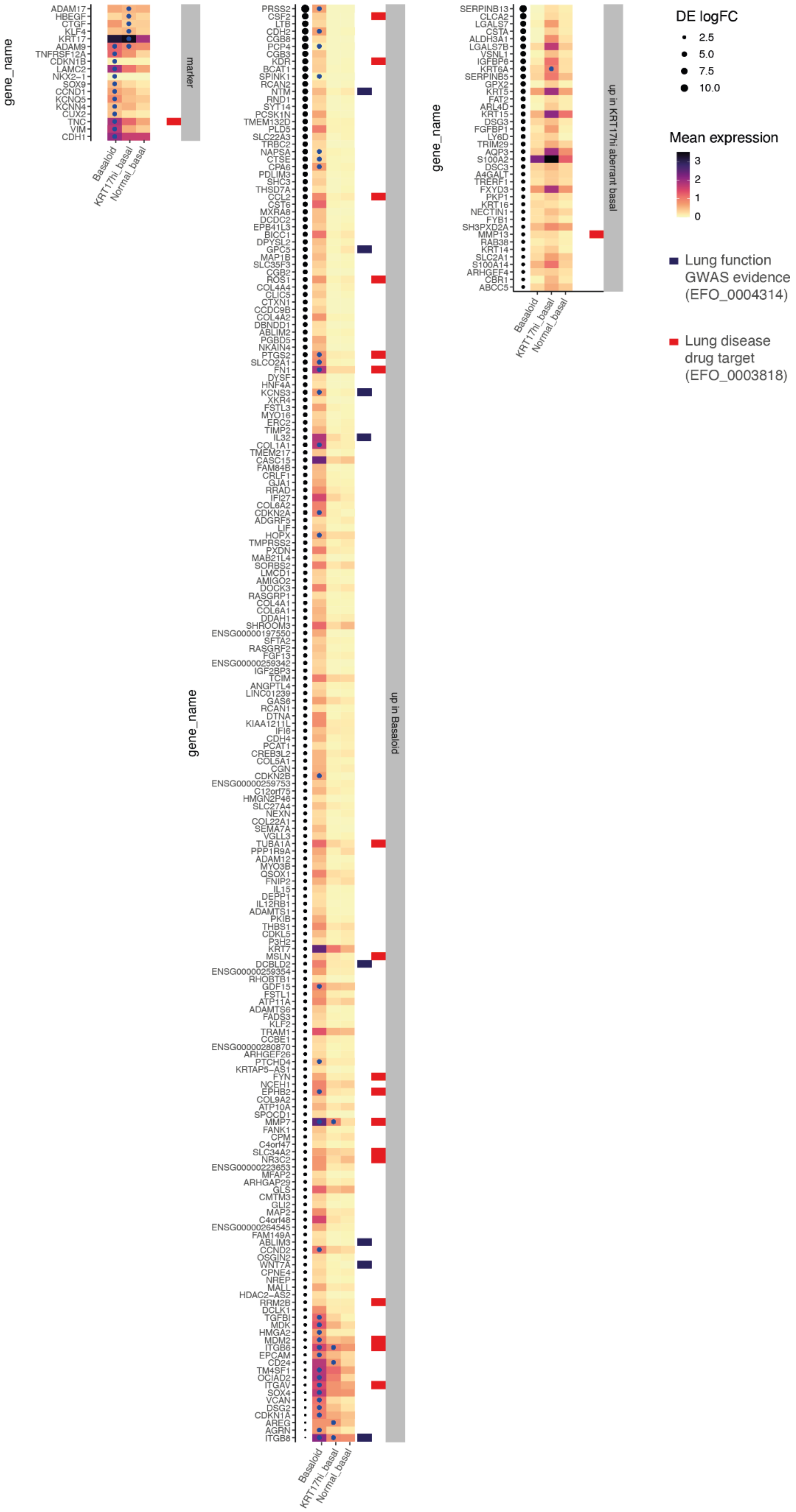
gene_name
ADAM17
HBEGF
CTGF
KLF4
KRT17
ADAM9
TNFRSF12A
CDKN1B
LAMC2
NKX2−1
SOX9
CCND1
KCNQ5
KCNN4
CUX2
TNC
VIM
CDH1
Basaloid
KRT17hi_basal
Normal_basal
marker
gene_name
PRSS2
CSF2
LTB
CDH2
CGB8
PCP4
CGB3
KDR
BCAT1
SPINK1
RCAN2
NTM
RND1
SYT14
PCSK1N
TMEM132D
PLD5
SLC22A3
TRBC2
NAPSA
CTSE
CPA6
PDLIM3
SHC3
THSD7A
CCL2
CST6
MXRA8
DCDC2
EPB41L3
BICC1
DPYSL2
GPC5
MAP1B
SLC35F3
CGB2
ROS1
COL4A4
CLIC5
CTXN1
CCDC9B
COL4A2
DBNDD1
ABLIM2
PGBD5
NKAIN4
PTGS2
SLCO2A1
FN1
DYSF
HNF4A
KCNS3
XKR4
FSTL3
MYO16
ERC2
TIMP2
IL32
COL1A1
TMEM217
CASC15
FAM84B
CRLF1
GJA1
RRAD
IFI27
COL6A2
CDKN2A
ADGRF5
LIF
HOPX
TMPRSS2
PXDN
MAB21L4
SORBS2
LMCD1
AMIGO2
DOCK3
RASGRP1
COL4A1
COL6A1
DDAH1
SHROOM3
ENSG00000197550
SFTA2
RASGRF2
FGF13
ENSG00000259342
IGF2BP3
TCIM
ANGPTL4
LINC01239
GAS6
RCAN1
DTNA
KIAA1211L
IFI6
CDH4
PCAT1
CREB3L2
COL5A1
CGN
CDKN2B
ENSG00000259753
C12orf75
HMGN2P46
SLC27A4
NEXN
COL22A1
SEMA7A
VGLL3
TUBA1A
PPP1R9A
ADAM12
MYO3B
QSOX1
FNIP2
IL15
DEPP1
IL12RB1
ADAMTS1
PKIB
THBS1
CDKL5
P3H2
KRT7
MSLN
DCBLD2
ENSG00000259354
RHOBTB1
GDF15
FSTL1
ATP11A
ADAMTS6
FADS3
KLF2
TRAM1
CCBE1
ENSG00000280870
ARHGEF26
PTCHD4
KRTAP5−AS1
FYN
NCEH1
EPHB2
COL9A2
ATP10A
SPOCD1
MMP7
FANK1
CPM
C4orf47
SLC34A2
NR3C2
ENSG00000223653
MFAP2
ARHGAP29
GLS
CMTM3
GLI2
MAP2
C4orf48
ENSG00000264545
FAM149A
ABLIM3
CCND2
OSGIN2
WNT7A
CPNE4
NREP
MAL
HDAC2−AS2
RRM2B
DCLK1
TGFBI
MDK
HMGA2
MDM2
ITGB6
EPCAM
CD24
TM4SF1
OCIAD2
ITGAV
SOX4
VCAN
DSG2
CDKN1A
AREG
AGRN
ITGB8
Basaloid
KRT17hi_basal
Normal_basal
up in Basaloid
gene_name
SERPINB13
CLCA2
LGALS7
CSTA
ALDH3A1
LGALS7B
VSNL1
IGFBP6
SERPINB5
GPX2
KRT5
FAT2
ARL4D
KRT15
DSG3
FGFBP1
LY6D
TRIM29
S100A2
DSC3
A4GALT
TRERF1
FXYD3
PKP1
KRT16
NECTIN1
FYB1
SH3PXD2A
MMP13
RAB38
KRT14
SLC2A1
S100A14
ARHGEF4
CBR1
ABCC5
Basaloid
KRT17hi_basal
Normal_basal
up in KRT17hi aberrant basal
DE logFC
2.5
5.0
7.5
10.0
Mean expression
3
2
1
0
Lung function GWAS evidence (EFO_0004314)
Lung disease drug target (EFO_0003818)

Supplementary figure *4: Differential expression analysis to identify markers for aberrant basal-like cells detected with ACR design.* *Heatmaps of mean expression of marker genes for aberrant basal-like phenotypes, identified by differential expression analysis between basaloid cells and KRT17hi aberrant basal cells (edgeR quasi-likelihood differential expression test [1]). DE genes were considered markers only if also significantly DE between the aberrant phenotype and normal basal cells (at 1% Benjamini-Hochberg FDR). Here we show marker genes that were expressed in at least 20% of cells of the aberrant phenotype, where log-Fold Change for DE was > 2 and with mean expression (log-normalized counts) > 0.2 (see suppl. table 4 for a full list of DE genes). Genes previously identified as markers of basaloid or KRT17hi aberrant basal cells are marked with a blue dot (the left facet shows expression of genes that were previously reported as markers, but that were not identified as DE between basaloid and aberrant KRT17hi basal in our analysis). Genes are ordered by absolute logFC, as indicated by points on the left. Genes significantly associated with GWAS variants for lung function (trait ID: EFO_0003892, using OpenTargets Genetics Locus2Gene association) are marked by a blue bar. Genes marked by a red bar on the right are validated target genes of drugs in trials for lung disease (trait ID: EFO_0003818, using OpenTargets database.*

2. Supplementary Notes

2.1 Alternatives to differential abundance analysis for OOR state detection.

In this study the workflow to detect disease-associated states relies on differential abundance analysis at the subpopulation level, using methods such as Milo [2], MELD [3], Co-varying neighbourhood analysis [4]. We recognize that scRNA-seq studies focused on disease-state identification, especially those using atlas datasets for this task [5–7], often omit differential abundance testing and define out-of-reference (OOR) cells using different criteria. In this note we test three alternative criteria on recovery of OOR states in our simulation framework: (a) Distance/segregation of OOR states in latent space, (b) query-mapping quality control metrics, and (c) differential expression analysis after label transfer. We discuss the limitations of these alternative approaches, and how they can also benefit from the use of control datasets.

2.1.1 Distance/segregation of OOR cells in latent space

Phenotypically distinct out-of-reference cells are generally expected to segregate from reference cells in the latent space, so that they could be distinguished by unsupervised clustering or other distance-based metric. Exploratory data analysis suggested that this is not always the case (see for example figure 2B: with AR design the OOR cells do not form a separate cluster).

To quantitatively test this hypothesis, we use the simulations with a single OOR state (figure 3A). For the latent space of each reference design(see Methods: Simulations Experiments - Latent space embedding), we train a KNN classifier to distinguish the OOR state in the disease dataset, using euclidean distance in the latent space (from scVI or scArches mapping) (k = 50). For each simulation with different OOR state, we split the disease dataset into a training (66% of cells) and test set (33% of cells) and measure precision and recall or OOR state classification on the test set. Of note, this experiment is a proof-of-concept to test whether OOR cells are indeed distant from in-reference cells in the latent space, but it does not represent a strategy to detect OOR cells in a discovery scenario, since it assumes prior labelling of true OOR cells.

In these experiments, the OOR cell state is not consistently distinguished by distance to reference cells in the latent space (Suppl. note figure 1). In general, the CR design with joint embedding performed best with this metric, but precision and recall are mostly dependent on the OOR cell population.

2.1.2 Mapping quality control metrics

In some cases different cell-level metrics for query-to-reference mapping quality are used to flag OOR cells, such as reconstruction error of the latent embedding model or uncertainty over a cell type classifier trained on the atlas dataset [5–7]. The assumption is that, if the disease-specific cell state was not seen during training of the latent embedding model, mapping quality metrics should be low.

We compare quality control (QC) metrics for scArches on the mapping of disease dataset onto the atlas dataset (mapping QC score), quantifying whether OOR cells and cell states are detectable by low QC score (i.e. higher uncertainty during query-to-reference mapping). For the label transfer uncertainty, we use the method presented by [6]. Briefly, we train a weighted KNN classifier for cell type labels on the latent embedding of the scVI reference dataset, setting the number of neighbors to k=100. For each cell in the query dataset, we extract its k nearest neighbors in the reference and the corresponding Euclidean distances, adjusted by a Gaussian kernel. We compute the probability $P(y|c)$ of assigning each label y to the query cell c by normalising across all adjusted distances. The label uncertainty corresponds to $1 - max_y(P(y|c))$.

For the reconstruction error metric, we take $s = 50$ samples from the posterior of the scArches model to generate profiles of predicted gene expression counts $\hat{x}_{i,c}$ for the highly variable genes used in training. We define the reconstructed gene expression profile $\bar{x}_c$ for each query cell c as:

$$\bar{x}_c = \frac{1}{s}\sum_{i=1}^{s} ln(\hat{x}_{i,c} + 1)$$

Given the true log-normalised gene expression profile for each cell x_c, we define the reconstruction error as the cosine distance between x_c and $\bar{x}_c$.

For both label uncertainty and reconstruction error, higher values correspond to lower mapping quality. To compare the mapping QC metrics to the differential abundance logFC on neighbourhoods defined with the ACR design, we calculate the average mapping QC score over all disease cells in the same neighbourhood.

In our hands, mapping quality metrics failed to distinguish ground-truth OOR cells (Suppl. note figure 2A) or neighbourhoods with high fraction of OOR cells (Suppl. note figure 2B).

We tested whether, by using an ACR design, we could increase the sensitivity by prioritising neighbourhoods where the mapping quality was worse for the disease dataset cells compared to the control dataset cells, taking the difference between average QC scores over disease cells and over control cells. This slightly improved OOR state detection performance using mapping QC metrics (Suppl. note figure 2C). However, overall we found that using mapping quality metrics to identify OOR populations performed significantly worse than using the logFC estimated from differential analysis.

Moreover, we compared these metrics also on real disease data, testing the ability to detect SPP1hi macrophages in the IPF dataset (Extended Data figure 7D-E). Here we confirmed that differential abundance analysis performed significantly better than the label transfer uncertainty score used in the HLCA study for detecting profibrotic macrophages [7].

2.1.3 OOR detection with label transfer and differential expression analysis

Finally, we reasoned that an atlas datasets might be employed for automatic label annotation, before identification of disease-specific cell populations through differential expression (DE) analysis between cells from disease and reference samples of each cell type.

To compare reference design with such a workflow, we tested whether OOR cell states in simulations could be distinguished by identifying over-expressed marker genes when running differential expression analysis on cell type clusters. For each simulation and reference design, we perform latent space embedding as described in the Methods and we train a KNN classifier to learn cell type annotation labels from the healthy reference latent embedding (k=50). We then predict annotations in the pseudo-disease dataset. We test for differential expression between donors in the healthy reference and pseudo-disease dataset for each cell type annotation, pseudo-bulking expression profiles by donor, with the edgeR quasi-likelihood test [1] using the implementation in the R package glmGamPoi [8], using a 10% FDR. For each cell type DE test, we remove genes expressed in less than 10 donors and with total counts below 20 and subsequently select the 10000 most highly variable genes, using the *modelGeneVar* function from the scran package [9]. We define a cell type cluster as disease-affected (i.e. misannotated cell population) if at least 5% of cells in the OOR state were predicted to belong to the cluster (suppl. note figure 3A). All other clusters are considered unaffected. For sensitivity analysis, we assume that no genes are differentially expressed in the unaffected clusters, while we test for the ability to detect differentially expressed marker genes of the OOR state in the affected cell state (suppl. note figure 3B). We define marker genes for each cell type using the top 50 predictive genes for each cell type based on a celltypist logistic regression model [10] trained on human immune cells.

Without a control dataset (AR design) we had a high rate of false positive genes in the unaffected clusters, where no DE genes are expected by design (suppl. note figure 3B). In contrast, we detected almost no false positives with designs where pseudo-disease dataset and control are contrasted in DE analysis, while still detecting a comparable rate of true positives (marker genes of the OOR state) (suppl. note figure 3B). We verified the rate of false positives is not attributable to failed label transfer (suppl. note figure 3C).

2.2 Robustness of OOR detection with ACR design

This note presents additional analyses on robustness of OOR state detection with ACR design to variation in the atlas dataset (see Results - Robustness of OOR detection with ACR design).

2.2.1 Leave-one-out analysis

We assessed whether one particular study amongst the 12 PBMC used to assemble the atlas dataset in simulations was driving the OOR detection performance with ACR design (see suppl. table 1 for the list of studies). To explore this, we performed repeated simulations with a leave-one-out approach. In each iteration, we excluded one of the 12 datasets from the analysis and then repeated the OOR state detection using the ACR design. For simulations with more than 400 OOR cells, the AUPRC of OOR state detection was stable when removing studies from the atlas dataset. The variability in performance increased where the OOR state population is smaller (< 400 cells) (suppl. note figure 4A). We did not observe systematically higher AUPRC with the largest atlas dataset (with all studies), and performance was not systematically lower when excluding a specific dataset (suppl. note figure 4B): in some cases we obtained the best performance when excluding data from the largest study (Powell dataset, comprising 1000 donors [11]). The variance in performance

with smaller OOR cell states likely reflects limitations in sensitivity of the current workflow for OOR state detection (Extended Data figure 4).

2.2.2 OOR state detection with cross-tissue atlas

In addition to varying the size and complexity of the atlas dataset, we also considered a scenario where the atlas does not perfectly match the cell types in the case-control dataset. Since large atlases are not yet available for all organs, we reasoned that in the absence of a tissue-specific atlas dataset case-control datasets could be mapped to large datasets collecting cell types from multiple tissues [10,12–14]. We applied our pseudo-disease simulation framework on a dataset of healthy lung cells from 30 donors, where we remove both immune and non-immune OOR states from the control (suppl. note figure 5A). Here we compared OOR detection performance using a tissue-specific lung cell atlas [7] or a cross-tissue atlas dataset [14], which does not include lung cells (suppl. note figure 5B).

Briefly, gene expression count matrices for human lung scRNA-seq data from Adams et al. 2020 were downloaded from GEO (GSE136831). As cell type annotations, we used uniform labels generated from integration of this dataset with the Human Lung Cell Atlas by Sikkema et al. [7], downloaded from Zenodo (https://zenodo.org/record/6337966). We subset the dataset to the control patients and to samples for which at least 1000 cells were captured, retaining data from 30 samples (libraries) from 30 donors. We further subsampled the dataset by randomly selecting 1000 cells per sample, to reduce the computational burden of this analysis. To simulate control and disease datasets for benchmarking, we split donors at random with equal probabilities into a disease subset (15 donors) and a control subset (15 donors). We checked that the split was balanced in overall cell state representation between the two sets with principal component analysis on cell type proportions for each sample. To simulate the presence of an out-of-reference cell state, we select one cell type label and remove all cells with that label from the control dataset (suppl. note figure 5A). We repeated this simulation with 6 annotated cell types in the Adams dataset, including 3 immune cell states (natural killer cells, dendritic cells of type 2, alveolar macrophages) and 3 non-immune/stromal cell states (fibroblasts, lymphatic endothelial cells and multiciliated epithelial cells). As a tissue-specific atlas, we used the core Human Lung Cell Atlas (HLCA) [7], collecting scRNA-seq data from 107 donors from 14 studies . We downloaded the scANVI model [15] trained on the core HLCA from Hugging Face[1] using the API available via scvi-tools v0.20.0. As a cross-tissue atlas, we used the Tabula Sapiens dataset [14], comprising scRNA-seq data from 24 tissues from 14 individuals. We downloaded the full dataset from figshare[2], excluding smart-seq2 libraries and data from lung samples. We trained an scVI model on this dataset as described above for the PBMC simulation, using donor ID as the batch covariate and training on 2000 highly variable genes to match the same number of genes used to train the HLCA model. For each simulated control/disease dataset assignment with different OOR population, for the ACR designs we performed latent embedding of the query dataset using scArches on the atlas models as described above. For the CR design with joint embedding, we trained an scVI model on the concatenated control and disease datasets as described for the PBMC dataset, using donor ID as the batch covariate and training on 2000 highly variable genes. We performed differential abundance analysis between the control and disease dataset with Milo, as described above.

[1] https://huggingface.co/scvi-tools/human-lung-cell-atlas
[2] https://figshare.com/articles/dataset/Tabula_Sapiens_release_1_0/14267219

We found OOR state detection performance with the two types of atlases was qualitatively comparable across simulations, with a slight decrease in sensitivity with the cross-tissue atlas in 2 of the 6 OOR cell states tested (suppl. note figure 5C-D). Notably, we observed the largest difference in sensitivity between the ACR design with a cross-tissue atlas and alternative designs in detection of out-of-reference multiciliated cells, which are a specialized class of epithelial cells found in the airways. Based on the maximum fraction of OOR cells in neighbourhoods for this simulation, multiciliated cells occupied neighbourhoods with other epithelial cell types with this design. These results suggest that using a cross-tissue atlas for contextualization of a case-control dataset might be a practical alternative where the availability of tissue-specific data might be scarce, although the power to detect changes in highly tissue-specialized subpopulations might decrease.

2.3 Matched controls allow the most sensitive out-of-reference detection

While our analyses suggest that mapping matched pseudo-disease and control datasets to an atlas dataset enhances the sensitivity to detect OOR states, in practice there are cases where collecting matched control samples is especially challenging (e.g. brain tumour studies, or studies of osteosarcoma). Therefore, we tested whether selecting a subset of samples from the atlas to use as control dataset in ACR design performed as well as using matched control samples. Specifically, we run the disease-state detection workflow with ACR design defining as control dataset either (a) donors from the same study (matched control), (b) a random subsample of donors from the atlas dataset (random control) or (c) a subset of donors selected by similarity in cell abundances to the disease dataset donors (close control). In particular, we applied the following unsupervised selection strategy, based on similarity between cell state proportions (illustrated in suppl. note figure 6A): we take latent dimensions learnt by training scVI on the atlas dataset and mapping the disease dataset with scArches (i.e. as described in Methods for AR design). In this latent space, we construct Milo neighbourhoods and the matrix of cell counts of dimension samples x neighbourhoods, as described in the Methods (section “Differential abundance analysis with Milo”). We log-normalise cell counts per sample (running *scanpy.pp.normalize_total* with parameters *target_sum=10000*, and *scanpy.pp.log1p*) and perform principal component analysis on the matrix of log-normalised cell counts. This generates a d-dimensional space (d=10) on which we compute Euclidean distances between disease and atlas samples. In this space, for each disease sample we take the closest atlas sample and we consider this set to be the “close controls”. If one atlas sample is the nearest neighbor to multiple disease dataset samples, we look at the second nearest neighbors for these samples and take those with the smallest Euclidean distance.

In all cases, the control donors are excluded from the atlas dataset used for scVI training. We found that using the matched control always outperformed subsampling from the atlas, and using the close control only marginally reduced the FDR compared to random subsampling (suppl. note figure 6B-C). Notably, using any type of control always outperformed differential analysis on the full atlas dataset.

These results highlight that comparison against a set of matched controls is always advantageous, likely minimizing false positives driven by hidden confounding factors. To an extent, accounting for confounding covariates in differential analysis based on available

metadata might reduce the false discovery rate when no matched controls are available. However, it is to be expected that comparing uniformly processed healthy and disease samples from a similar population will minimise variability associated with known and hidden confounders. Selecting samples from published data based on phenotypic similarity led to a small improvement in performance compared to selection of samples at random. While here we applied a naive approach to define sample similarity, novel methods to model sample-level heterogeneity in scRNA-seq data are being explored [16–18]. These could improve the matching of disease samples to optimal controls, and provide new insights into which technical and demographic variables are likely to affect disease-to-healthy comparisons.

2.4 Impact of latent embedding strategy on OOR state detection

Throughout this study, we use a transfer learning (TL) method for joint dimensionality reduction with an atlas dataset [6], as an efficient alternative to joint embedding on concatenated datasets. We expect our reference design analysis results to be robust to the use of other TL-based methods for query-to-reference mapping [5,19]. In the COVID-19 analysis we observed minimal changes to the embedding with ACR design when using *de novo* integration instead of TL (figure 5D). Conversely, for the reference design without an atlas (CR design) we observed differences for the TL and joint embedding, which was not surprising given the limitations of TL approaches when comparable number of cells are in the reference dataset (control) and in the query dataset (disease) [6]. We found that the CR design with joint embedding significantly increases the performance for disease-state detection compared to CR design with TL (figure 3B, figure 5D), although this approach appears to be highly sensitive to feature selection (figure 3C). Of note, in this study we used a standard approach to select features for integration, choosing the default method implemented in the most popular single-cell analysis pipelines [20,21]. Many studies use more complex feature selection strategies that might be batch-aware or tissue-specific (e.g. excluding known “soup” genes). Given the wealth of options, we believe robustness of workflows to feature selection choices is a key feature to consider. To date, integration methods and parameters, including feature selection, have been primarily benchmarked on the tasks of batch correction, automatic cell type annotation and fast analysis of new data generated from the same biological condition [22–24]. While the systematic comparison of different integration strategies on disease datasets is beyond the scope of this study, the benchmarking set-up and python package presented in this study (https://github.com/MarioniLab/oor_benchmark) could serve as basis for future comparisons focused on alternative integration methods.

3. Supplementary Note Figures

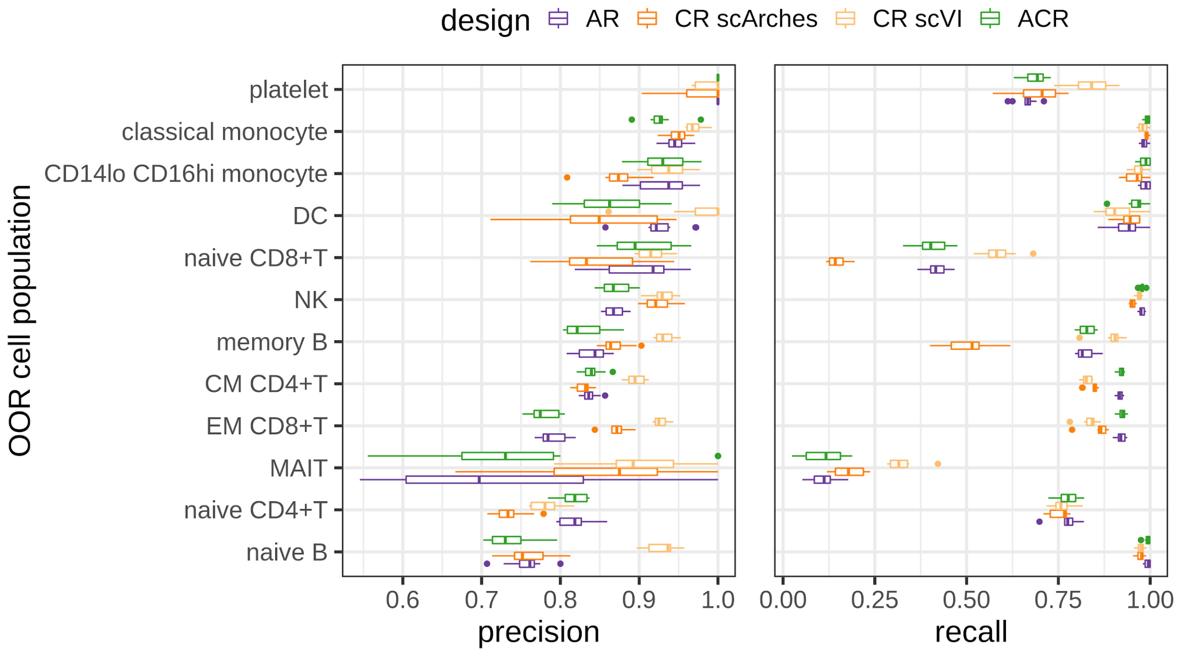

Supplementary note figure 1: ***The out-of-reference state is not always distinguished by position in the latent space after scArches mapping.*** *Estimated precision (left) and recall (right) for simulations with different OOR cell populations (y-axis) when training a KNN classifier on the latent space of disease cells to distinguish the true OOR cells. Each box plot shows the median and interquartile range for 10 classifiers trained with a different split of cells into training and test set (n = 10 classifiers for each design and OOR cell population). Precision and recall on the test set are shown. This analysis was performed only on simulations where at least 100 cells were found in the OOR state in the disease dataset. In boxplots the center line denotes the median; box limits, first and third quartiles; whiskers, 1.5X interquartile range*

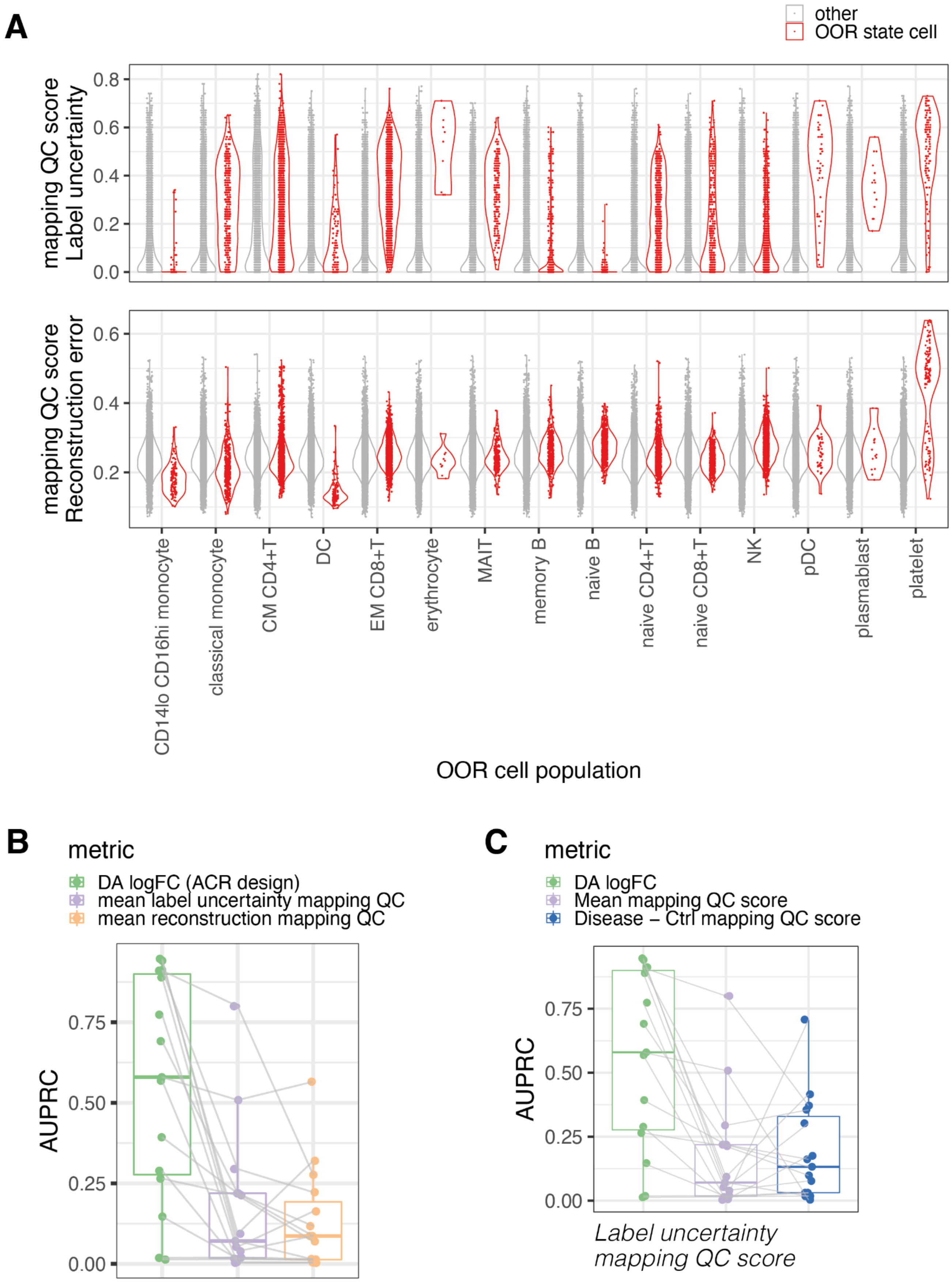
A
other
OOR state cell
mapping QC score
Label uncertainty
0.8
0.6
0.4
0.2
0.0
mapping QC score
Reconstruction error
0.6
0.4
0.2
CD14lo CD16hi monocyte
classical monocyte
CM CD4+T
DC
EM CD8+T
erythrocyte
MAIT
memory B
naive B
naive CD4+T
naive CD8+T
NK
pDC
plasmablast
platelet
OOR cell population
B
metric
DA logFC (ACR design)
mean label uncertainty mapping QC
mean reconstruction mapping QC
AUPRC
0.75
0.50
0.25
0.00
C
metric
DA logFC
Mean mapping QC score
Disease – Ctrl mapping QC score
AUPRC
0.75
0.50
0.25
0.00
Label uncertainty
mapping QC score

Supplementary note figure 2*: OOR state detection using to query-to-reference mapping quality control metrics.* *(A)* *Violin plots of mapping QC scores for cells in OOR state (red) and all other cells (grey) across 15 simulations with different OOR cell populations (x-axis). The top plot shows label uncertainty as mapping QC score, the bottom plot shows reconstruction error as mapping QC score.* ***(B)*** *Boxplot of Area Under the Precision-Recall Curve (AUPRC) for detection of OOR state neighbourhoods with 3 different metrics: differential abundance log-Fold Change with ACR design (DA logFC, green), mean label uncertainty (purple), mean reconstruction error (orange). Points represent simulations with different OOR populations (n = 15 simulations). Tests on the same simulated data are connected.* ***(C)*** *As in (B), but we compare DA logFC and mean label uncertainty to the difference between mean label uncertainty of disease cells and mean label uncertainty of control cells in the same neighbourhood (blue). In boxplots the center line denotes the median; box limits, first and third quartiles; whiskers, 1.5X interquartile range.*

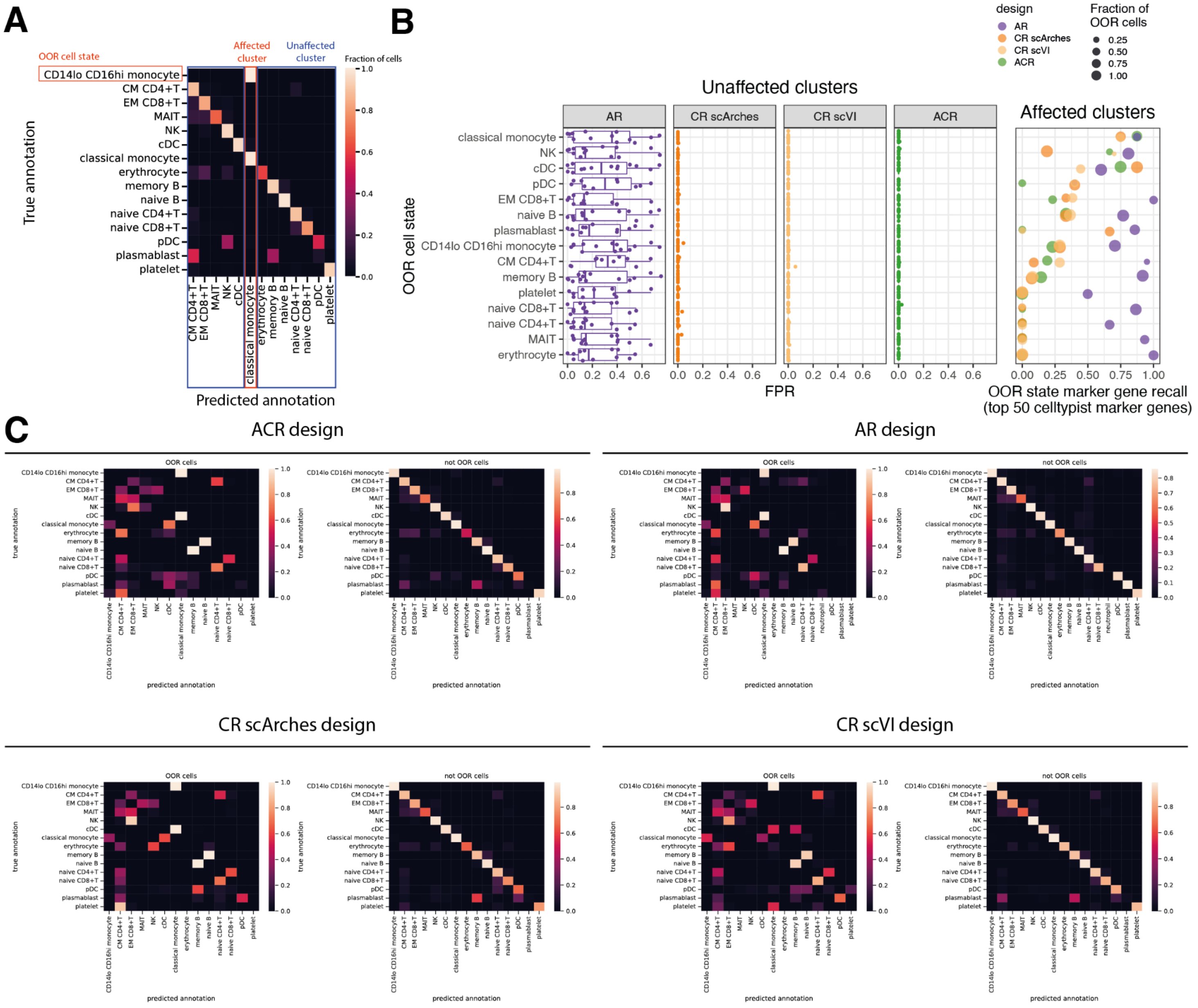

Supplementary note figure 3: Out-of-reference state detection with label transfer and differential expression analysis. *(**A**) Example outcome of label transfer from healthy reference to pseudo-disease dataset: heatmap of confusion matrix of true (y-axis) versus predicted annotations (x-axis) from training a KNN classifier to distinguish healthy reference labels from the latent embedding space. The heatmap colours are proportional to the fraction of cells with each predicted label. In this simulation, the CD14lo CD16hi monocytes were the out-of-reference state, which were mislabelled as classical monocytes with label transfer. We consider the cluster with the highest fraction of cells from the OOR state to be the affected cluster, where we compute OOR state marker gene recall. The rest of the clusters are considered unaffected, where no differentially expressed genes are expected. (**B**) (left) False Positive Rate (FPR) for detection of differentially expressed genes in unaffected clusters: each boxplot represents a simulation with a different OOR cell state removed, each point represents a cell type cluster in which the DE test was performed (n = 15 cell type clusters). Results are grouped by reference design (facets, colours). In boxplots the center line denotes the median; box limits, first and third quartiles; whiskers, 1.5X interquartile range; (right) Recall (true positive rate) of marker genes for the OOR state in the affected cluster. Marker genes were defined using a CellTypist model trained on immune cells [10]. Points are coloured by reference design. Point size is proportional to the fraction of OOR cells in the affected cluster. (**C**) For each reference design, confusion matrices for label transfer results for cells in OOR state (left) and all other cells (right) across all simulations with different OOR cell states.*

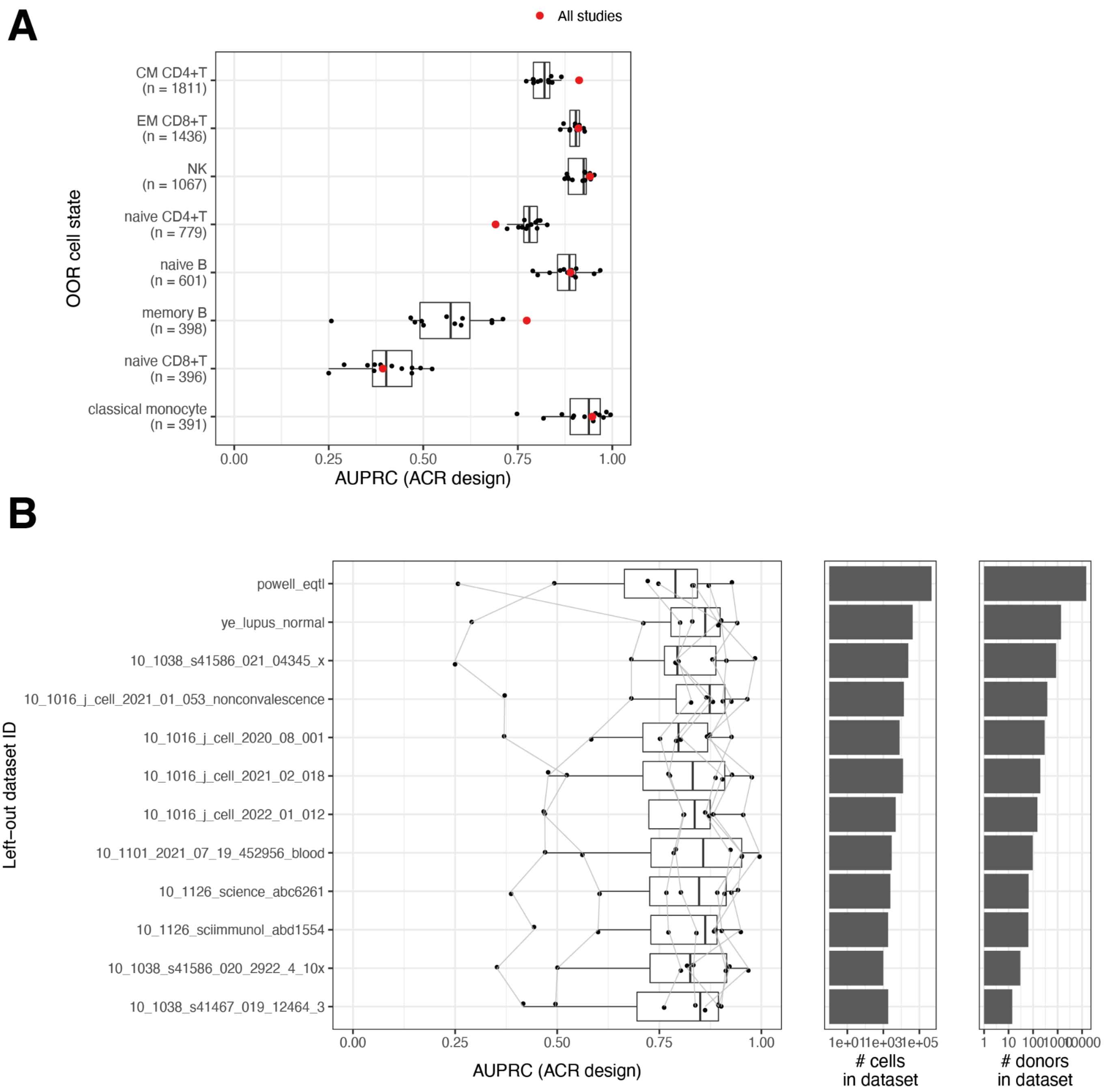

Supplementary note figure 4: Precision-recall analysis on ACR design with leave-one-out cross validation on atlas dataset. *(**A**) Area under the precision-recall curve (AUPRC, x-axis) for OOR detection with ACR design of simulations with different OOR states (y-axis) where one of the 12 PBMC datasets was excluded from the atlas reference dataset. The red dot denotes the performance of OOR detection when using all datasets. The number of cells in the OOR cell state is indicated on the y-axis. (**B**) AUPRC values as in (A), but simulations are grouped by ID of the excluded dataset (n = 8 simulations with different OOR states). The barplots to the right indicate the size of the removed dataset in terms of cell numbers and donor numbers. Simulations with the same OOR cell state are connected by a grey line. In boxplots the center line denotes the median; box limits, first and third quartiles; whiskers, 1.5X interquartile range.*

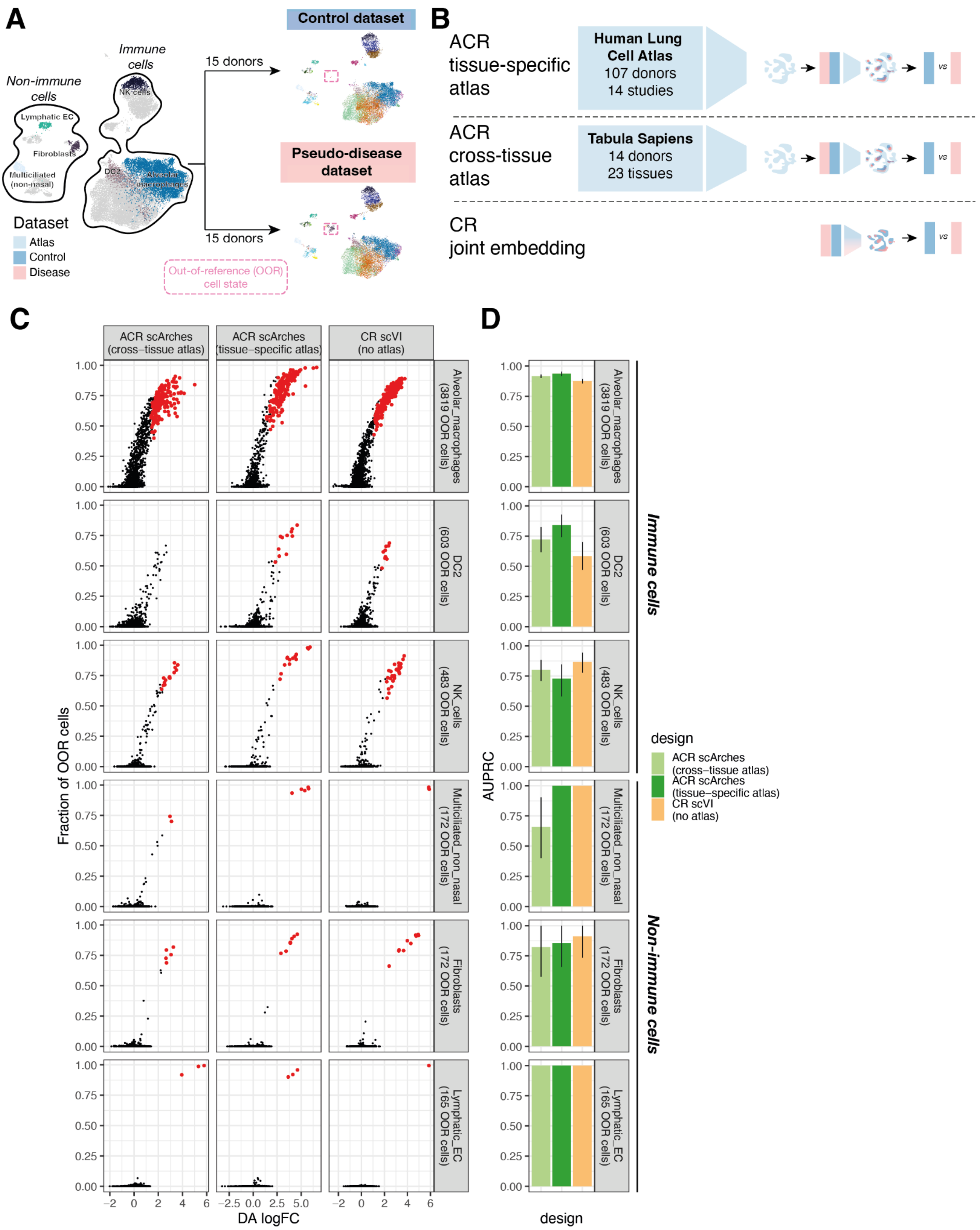
A
Control dataset
Immune cells
Non-immune cells
15 donors
NK cells
Lymphatic EC
Fibroblasts
DC2
Alveolar macrophages
Multiciliated (non-nasal)
Pseudo-disease dataset
Dataset
Atlas
Control
Disease
15 donors
Out-of-reference (OOR) cell state
B
ACR tissue-specific atlas
Human Lung Cell Atlas
107 donors
14 studies
ACR cross-tissue atlas
Tabula Sapiens
14 donors
23 tissues
CR joint embedding
vs
C
ACR scArches (cross–tissue atlas)
ACR scArches (tissue–specific atlas)
CR scVI (no atlas)
Alveolar_macrophages (3819 OOR cells)
DC2 (603 OOR cells)
NK_cells (483 OOR cells)
Multiciliated_non_nasal (172 OOR cells)
Fibroblasts (172 OOR cells)
Lymphatic_EC (165 OOR cells)
Fraction of OOR cells
DA logFC
D
AUPRC
design
Immune cells
Non-immune cells
design
ACR scArches (cross–tissue atlas)
ACR scArches (tissue–specific atlas)
CR scVI (no atlas)

Supplementary note figure 5: Reference design comparison with tissue-specific or cross-tissue atlas on human lung cells. *(A) Overview of the human lung parenchyma cells used for OOR simulation. Data from 30 healthy donors from Adams et al. 2020 were split in two groups to simulate control dataset and pseudo-disease dataset. In each simulation, one of 6 cell type cell populations were removed from the control dataset to simulate the presence of an out-of-reference (OOR) population. The cell type populations used as OOR cells are coloured in the UMAP embedding. (B) Schematic of the atlas datasets used for reference design comparison. Data from lung samples was excluded from the Tabula Sapiens dataset. (C) Scatterplot of differential abundance log-Fold Change (DA logFC) against fraction of out-of-reference (OOR) cells for each neighbourhood, in simulations with different OOR cell populations (indicated in y-axis). Coloured points indicate neighbourhoods where the enrichment was significant (10% SpatialFDR, logFC > 0). (D) Barplots of area under the precision-recall curve estimated for each reference design in simulations with different OOR cell populations. Error bars denote the 95% confidence interval of AUPRC calculated from bootstrapping with 1000 resamplings. The height of the bar denotes the AUPRC computed on the real data.*

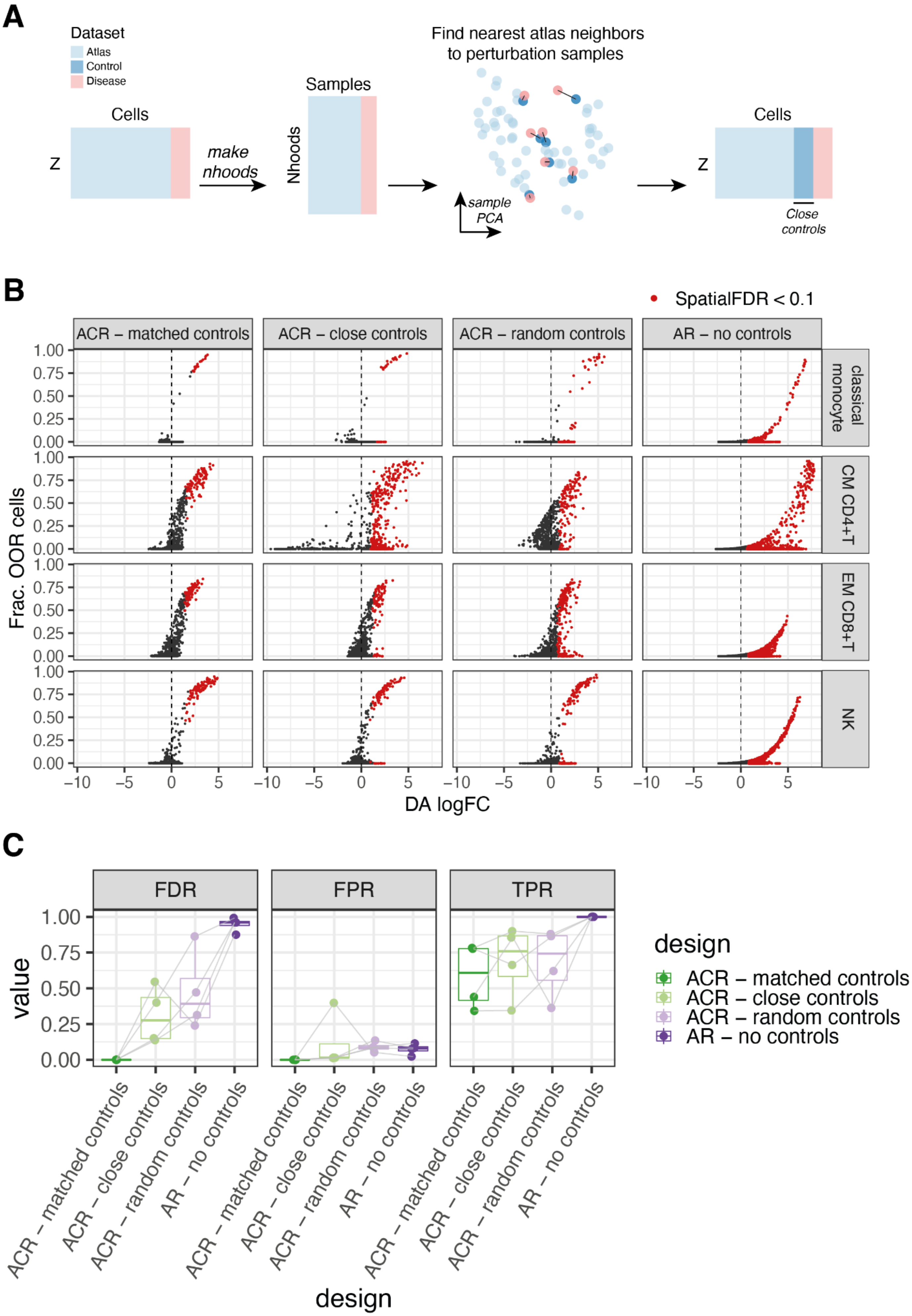
A
Dataset
Atlas
Control
Disease
Cells
Z
make nhoods
Samples
Nhoods
Find nearest atlas neighbors to perturbation samples
sample PCA
Cells
Z
Close controls
B
SpatialFDR < 0.1
ACR – matched controls
ACR – close controls
ACR – random controls
AR – no controls
classical monocyte
CM CD4+T
EM CD8+T
NK
Frac. OOR cells
DA logFC
C
FDR
FPR
TPR
value
design
design
ACR – matched controls
ACR – close controls
ACR – random controls
AR – no controls

Supplementary note figure 6*: Comparison of matched controls and controls selected from the atlas dataset.* ***(A)*** *Schematic of method used for unsupervised selection of "close controls" from the atlas samples: the latent space (Z) learnt from mapping of the disease dataset to the atlas dataset was used to construct a KNN graph and neighbourhoods. We generated a count matrix storing the number of cells from each sample in each neighbourhood (as used in Milo differential abundance analysis). We projected samples over principal components (PCs) that explain the highest variance in the neighbourhood count matrix and we computed euclidean distances between disease and atlas samples in the top 10 PCs. For each disease sample we take the closest atlas sample and we consider this set of samples to be the "close control" dataset.* ***(B)*** *Scatterplot of differential abundance log-Fold Change (DA logFC) against fraction of out-of-reference (OOR) cells for each neighbourhood, in simulations with different unseen cell populations (rows), with different control samples used for ACR design or no controls (AR design). Colored points indicate neighbourhoods where the enrichment was significant (10% SpatialFDR and logFC > 0).* ***(C)*** *Quantitative comparison of performance with different control samples used for ACR design or no controls (AR design) in detection of OOR cell states (neighbourhoods where the fraction of cells from the perturbation-specific state is higher than 20% of the maximum fraction). Each point represents a simulation with different OOR cell states (n = 4 simulations, as shown in B). To compare performance considering the logFC and confidence (10% SpatialFDR), we measured the false discovery rate (FDR), false positive rate (FPR) and true positive rate (TPR). Tests on the same simulated data are connected. In boxplots the center line denotes the median; box limits, first and third quartiles; whiskers, 1.5X interquartile range.*

Supplementary Note References

1. Robinson MD, Oshlack A. A scaling normalization method for differential expression analysis of RNA-seq data. Genome Biol. 2010;11: R25.

2. Dann E, Henderson NC, Teichmann SA, Morgan MD, Marioni JC. Differential abundance testing on single-cell data using k-nearest neighbor graphs. Nat Biotechnol. 2022;40: 245–253.

3. Burkhardt DB, Stanley JS 3rd, Tong A, Perdigoto AL, Gigante SA, Herold KC, et al. Quantifying the effect of experimental perturbations at single-cell resolution. Nat Biotechnol. 2021;39: 619–629.

4. Reshef YA, Rumker L, Kang JB, Nathan A, Korsunsky I, Asgari S, et al. Co-varying neighborhood analysis identifies cell populations associated with phenotypes of interest from single-cell transcriptomics. Nat Biotechnol. 2022;40: 355–363.

5. Hao Y, Hao S, Andersen-Nissen E, Mauck WM 3rd, Zheng S, Butler A, et al. Integrated analysis of multimodal single-cell data. Cell. 2021;184: 3573–3587.e29.

6. Lotfollahi M, Naghipourfar M, Luecken MD, Khajavi M, Büttner M, Wagenstetter M, et al. Mapping single-cell data to reference atlases by transfer learning. Nat Biotechnol. 2022;40: 121–130.

7. Sikkema L, Strobl D, Zappia L, Madissoon E, Markov NS, Zaragosi L, et al. An integrated cell atlas of the human lung in health and disease. bioRxiv. 2022. p. 2022.03.10.483747. doi:10.1101/2022.03.10.483747

8. Ahlmann-Eltze C, Huber W. glmGamPoi: fitting Gamma-Poisson generalized linear models on single cell count data. Bioinformatics. 2021;36: 5701–5702.

9. Lun ATL, McCarthy DJ, Marioni JC. A step-by-step workflow for low-level analysis of single-cell RNA-seq data with Bioconductor. F1000Research. 2016. p. 2122. doi:10.12688/f1000research.9501.2

10. Domínguez Conde C, Xu C, Jarvis LB, Rainbow DB, Wells SB, Gomes T, et al. Cross-tissue immune cell analysis reveals tissue-specific features in humans. Science. 2022;376: eabl5197.

11. Yazar S, Alquicira-Hernandez J, Wing K, Senabouth A, Gordon MG, Andersen S, et al. Single-cell eQTL mapping identifies cell type–specific genetic control of autoimmune disease. Science. 2022;376: eabf3041.

12. Eraslan G, Drokhlyansky E, Anand S, Fiskin E, Subramanian A, Slyper M, et al. Single-nucleus cross-tissue molecular reference maps toward understanding disease gene function. Science. 2022;376: eabl4290.

13. Suo C, Dann E, Goh I, Jardine L, Kleshchevnikov V, Park J-E, et al. Mapping the developing human immune system across organs. Science. 2022;376: eabo0510.

14. Tabula Sapiens Consortium*, Jones RC, Karkanias J, Krasnow MA, Pisco AO, Quake SR, et al. The Tabula Sapiens: A multiple-organ, single-cell transcriptomic atlas of humans. Science. 2022;376: eabl4896.

15. Xu C, Lopez R, Mehlman E, Regier J, Jordan MI, Yosef N. Probabilistic harmonization

and annotation of single-cell transcriptomics data with deep generative models. Mol Syst Biol. 2021;17: e9620.

16. Chen WS, Zivanovic N, van Dijk D, Wolf G, Bodenmiller B, Krishnaswamy S. Uncovering axes of variation among single-cell cancer specimens. Nat Methods. 2020;17: 302–310.

17. Boyeau P, Hong J, Gayoso A, Jordan MI, Azizi E, Yosef N. Deep generative modeling for quantifying sample-level heterogeneity in single-cell omics. bioRxiv. 2022. p. 2022.10.04.510898. doi:10.1101/2022.10.04.510898

18. Mitchel J, Grace Gordon M, Perez RK, Biederstedt E, Bueno R, Ye CJ, et al. Tensor decomposition reveals coordinated multicellular patterns of transcriptional variation that distinguish and stratify disease individuals. bioRxiv. 2022. p. 2022.02.16.480703. doi:10.1101/2022.02.16.480703

19. Kang JB, Nathan A, Weinand K, Zhang F, Millard N, Rumker L, et al. Efficient and precise single-cell reference atlas mapping with Symphony. Nat Commun. 2021;12: 5890.

20. Satija R, Farrell JA, Gennert D, Schier AF, Regev A. Spatial reconstruction of single-cell gene expression data. Nat Biotechnol. 2015;33: 495–502.

21. Wolf FA, Angerer P, Theis FJ. SCANPY: large-scale single-cell gene expression data analysis. Genome Biol. 2018;19: 15.

22. Tran HTN, Ang KS, Chevrier M, Zhang X, Lee NYS, Goh M, et al. A benchmark of batch-effect correction methods for single-cell RNA sequencing data. Genome Biol. 2020;21: 12.

23. Luecken MD, Büttner M, Chaichoompu K, Danese A, Interlandi M, Mueller MF, et al. Benchmarking atlas-level data integration in single-cell genomics. Nat Methods. 2022;19: 41–50.

24. Chazarra-Gil R, van Dongen S, Kiselev VY, Hemberg M. Flexible comparison of batch correction methods for single-cell RNA-seq using BatchBench. Nucleic Acids Research. 2021. doi:10.1093/nar/gkab004
