# Supplementary material for: Precise identification of cell states altered in disease using healthy single-cell references
Source: Nat Genet. 2023 Oct 12;55(11):1998–2008. doi: 10.1038/s41588-023-01523-7 (PMC10632138; doi:10.1038/s41588-023-01523-7)
Supplement: Supplementary file 2 — Reporting Summary [file 41588_2023_1523_MOESM2_ESM.pdf]

nature portfolio

Corresponding author(s): John C. Marioni, Sarah A. Teichmann

Last updated by author(s): Aug 10, 2023

Reporting Summary

Nature Portfolio wishes to improve the reproducibility of the work that we publish. This form provides structure for consistency and transparency in reporting. For further information on Nature Portfolio policies, see our Editorial Policies and the Editorial Policy Checklist.

Statistics

For all statistical analyses, confirm that the following items are present in the figure legend, table legend, main text, or Methods section.

n/a	Confirmed	
☐	☒	The exact sample size (*n*) for each experimental group/condition, given as a discrete number and unit of measurement
☐	☒	A statement on whether measurements were taken from distinct samples or whether the same sample was measured repeatedly
☐	☒	The statistical test(s) used AND whether they are one- or two-sided<br>*Only common tests should be described solely by name; describe more complex techniques in the Methods section.*
☐	☒	A description of all covariates tested
☐	☒	A description of any assumptions or corrections, such as tests of normality and adjustment for multiple comparisons
☐	☒	A full description of the statistical parameters including central tendency (e.g. means) or other basic estimates (e.g. regression coefficient) AND variation (e.g. standard deviation) or associated estimates of uncertainty (e.g. confidence intervals)
☐	☒	For null hypothesis testing, the test statistic (e.g. *F*, *t*, *r*) with confidence intervals, effect sizes, degrees of freedom and *P* value noted<br>*Give P values as exact values whenever suitable.*
☒	☐	For Bayesian analysis, information on the choice of priors and Markov chain Monte Carlo settings
☒	☐	For hierarchical and complex designs, identification of the appropriate level for tests and full reporting of outcomes
☐	☒	Estimates of effect sizes (e.g. Cohen's *d*, Pearson's *r*), indicating how they were calculated

Our web collection on statistics for biologists contains articles on many of the points above.

Software and code

Policy information about availability of computer code

Data collection	Python packages: python v3.10.6, anndata v0.8.0
Data analysis	Python packages: python v3.10.6, milopy v0.1.0, scanpy v1.9.1, anndata v0.8.0, scvi-tools v0.17.4 (scvi-tools v0.20.0 for access to trained Human Lung Cell Atlas model), sklearn v0.0.post1, meld v1.0.0, cna v0.1.4<br>R packages: R v4.0.5, bioconductor-edger v3.32.1 bioconductor-scran v1.28.2<br><br>Links to custom code repositories can be found in the "Code availability' section.

For manuscripts utilizing custom algorithms or software that are central to the research but not yet described in published literature, software must be made available to editors and reviewers. We strongly encourage code deposition in a community repository (e.g. GitHub). See the Nature Portfolio guidelines for submitting code & software for further information.

Data

Policy information about availability of data

All manuscripts must include a data availability statement. This statement should provide the following information, where applicable:

- Accession codes, unique identifiers, or web links for publicly available datasets
- A description of any restrictions on data availability
- For clinical datasets or third party data, please ensure that the statement adheres to our policy

All the data used for analysis is publicly available.
Simulation study and COVID-19 analysis: Data used in the simulation study was downloaded from the cellxgene portal (https://cellxgene.cziscience.com/collections) (see Suppl Table 1 for dataset IDs for all studies)
IPF analysis:
- IPF and control data was downloaded from the Gene Expression Omnibus (GSE136831)
- Human Lung Cell Atlas model and data was downloaded from Zenodo (https://zenodo.org/record/6337966)
- Information about drug targets for lung disease was downloaded from OpenTargets Platform (https://platform.opentargets.org/) (trait ID: EFO_0003818). The downloaded table is available at https://github.com/MarioniLab/oor_design_reproducibility/blob/master/metadata/opentargets_drugs.EFO_0003818.tsv
- Information about genes with genetic association to lung function was downloaded from OpenTargets Genetics Platform (https://genetics.opentargets.org/) (trait ID: EFO_0004314). The downloaded table is available at http://github.com/MarioniLab/oor_design_reproducibility/blob/master/metadata/opentargets_genetics.EFO_0004314.csv

Processed data objects and trained scVI models are available via figshare (https://doi.org/10.6084/m9.figshare.21456645). Additional metadata is shared in the reproducibility repository (https://github.com/MarioniLab/oor_design_reproducibility).

Research involving human participants, their data, or biological material

Policy information about studies with human participants or human data. See also policy information about sex, gender (identity/presentation), and sexual orientation and race, ethnicity and racism.

Reporting on sex and gender	Sex- and gender-based analysis was not performed.
Reporting on race, ethnicity, or other socially relevant groupings	No human research participants were recruited for this study.
Population characteristics	No human research participants were recruited for this study.
Recruitment	No human research participants were recruited for this study.
Ethics oversight	No human research participants were recruited for this study.

Note that full information on the approval of the study protocol must also be provided in the manuscript.

Field-specific reporting

Please select the one below that is the best fit for your research. If you are not sure, read the appropriate sections before making your selection.

☒ Life sciences ☐ Behavioural & social sciences ☐ Ecological, evolutionary & environmental sciences

For a reference copy of the document with all sections, see nature.com/documents/nr-reporting-summary-flat.pdf

Life sciences study design

All studies must disclose on these points even when the disclosure is negative.

Sample size	No sample-size calculation was performed a priori. Analysis involved thousands of cells from hundreds of individuals per comparison, providing a robust sample size, in line with similar scRNA-seq comparisons.
Data exclusions	Samples profiled with Smart-seq-2 protocol in the Stephenson et al. dataset were excluded (3 samples) to avoid batch effects with 10X genomics data.
Replication	Findings from simulation experiments were replicated changing several parameters (including size of atlas/control/disease dataset, type of out-of-reference cell state, number of out-of-reference cell states), as detailed in the "Robustness of OOR detection with ACR design" section of results. Findings on disease-specific cell types were not replicated in independent datasets due to limited availability of atlas and disease scRNA-seq datasets.
Randomization	In simulation studies, studies and donors were split into atlas, disease and control datasets at random.

Blinding	Our analysis consisted on computational method benchmarking and exploratory data analysis where blinding is not possible and quantitative outcomes are not dependent on blinding.

Reporting for specific materials, systems and methods

We require information from authors about some types of materials, experimental systems and methods used in many studies. Here, indicate whether each material, system or method listed is relevant to your study. If you are not sure if a list item applies to your research, read the appropriate section before selecting a response.

Materials & experimental systems

n/a	Involved in the study
☒	☐ Antibodies
☒	☐ Eukaryotic cell lines
☒	☐ Palaeontology and archaeology
☒	☐ Animals and other organisms
☒	☐ Clinical data
☒	☐ Dual use research of concern
☒	☐ Plants

Methods

n/a	Involved in the study
☒	☐ ChIP-seq
☒	☐ Flow cytometry
☒	☐ MRI-based neuroimaging
